# Comprehensive analysis of the expression of SLC30A family genes and prognosis in human gastric cancer

**DOI:** 10.1038/s41598-020-75012-w

**Published:** 2020-10-27

**Authors:** Yongdong Guo, Yutong He

**Affiliations:** Cancer Institute, Fourth Hospital of Hebei Medical University, Shijiazhuang, 050011 China

**Keywords:** Cancer genetics, Gastrointestinal cancer, Oncogenes

## Abstract

The solute carrier 30 (SLC30) family genes play a fundamental role in various cancers. However, the diverse expression patterns, prognostic value, and potential mechanism of SLC30A family genes in gastric cancer (GC) remain unknown. Herein, we analyzed the expression and survival data of SLC30A family genes in GC patients using multiple bioinformatic approaches. Expression data of SLC30A family genes for GC patients were extracted from the Cancer Genome Atlas (TCGA) and genetic alteration frequency assessed by using cBioportal database. And validated the expression of SLC30A family genes in GC tissues and corresponding normal tissues. The prognostic value of SLC30A family genes in gastric cancer patients were explored using Kaplan–Meier plotter database. Functional enrichment analysis performed using DAVID database and clusterProfiler package. And ssGSEA algorithm was performed to explore the relationship between the SLC30A family genes and the infiltration of immune cells. We found that the median expression levels of *SLC30A1-3, 5–7, and 9* were significantly upregulated in gastric cancer tissues compared to non-cancerous tissues, while *SLC30A4* was downregulated. Meanwhile, *SLC30A1-7*, and *9* were significantly correlated with advanced tumor stage and nodal metastasis status, *SLC30A5-7,* and *9–10* were significantly related to the *Helicobacter pylori* infection status of GC patients. High expression of five genes (*SLC30A1, 5–7,* and *9*) was significantly correlated with better overall survival (OS), first progression survival (FPS), and post progression survival (PPS). Conversely, upregulated *SLC30A2-4*, *8*, and *10* expression was markedly associated with poor OS, FP and PPS. And SLC30A family genes were closely associated with the infiltration of immune cells. The present study implied that *SLC30A5* and *7* may be potential biomarkers for predicting prognosis in GC patients, *SLC30A2* and *3* play an oncogenic role in GC patients and could provide a new strategy for GC patients treatment.

## Background

Gastric cancer (GC) is one of the most prevalent malignancy worldwide^1^. According to the latest cancer statistics, GC is considered the second most common cause of cancer-related mortality in the world^2^. Most GC is induced by a complex interaction between *Helicobacter pylori* and host factors^3^. Multiple studies have reported that various environmental elements are considered as gastric cancer risk factors including trace elements^4–6^. Surgery is the primary therapeutic for GC patients, even with the advances in diagnosis and treatment in the past few years. GC patient prognosis remains unfavorable in that many patients are still initially diagnosed at an advanced stage^7^. Hence, it is extremely important to seek potential prognostic biomarkers for early diagnosis and novel therapeutic targets.

Cixian and Linxian, located in northern China along the Taihang Mountain chain, are one of the higher-risk areas for upper gastrointestinal cancer both in China and worldwide^8–11^ (Supplementary Figures S1-2). Previous studies showed that individuals living in Cixian and Linxian have a zinc intake below the recommended daily allowance and higher incidence and mortality rates of GC than that of other regions^9,10,12^. For zinc to perform its various bioactive roles, many specific systems to transport zinc across the biological membrane are needed^13^. Therefore, zinc transport proteins are indispensable for facilitating the bioactive roles of zinc. Zinc homeostasis is mostly maintained by the Zn transporter (SLC30A , ZnT) and Irt-related proteins (SLC39A, ZIP), which play critical roles in a wide array of biological processes and cellular functions including growth, endocrine, reproductive, and immune processes^14–16^.

Emerging evidence indicates that the solute carrier (SLC) 39A family of genes, also known as zinc importer genes, are significantly correlated with prognosis in GC patients^17^. Therefore, we hypothesized that SLC30A family genes, also known as zinc exporter genes, might also be strongly associated with GC. The SLC30A family, including SLC30A1-10, contribute to the cytoplasmic zinc balance by exporting zinc to the extracellular space or moving cytoplasmic zinc into intracellular compartments when cellular zinc levels are elevated^16^. Furthermore, multiple studies have revealed that SLC30A family genes are dysregulated and played a critical role in various kinds of cancer, including pancreatic cancer^18^, invasive breast ductal carcinoma^18^, and esophageal cell carcinoma^20^. Previous studies have reported that *SLC30A1*, *9* and *10* were significantly upregulated in prostate cancer tissues compared to normal tissues, while *SLC30A5*-*6* were strongly downregulated^21–23^. Upregulated *SLC30A5-7* expression might play a critical role in coordinating transcriptional programming associated with the increased activity of the early secretory pathway in colorectal cancer^24^. Nevertheless, the functional and prognostic significance of SLC30A family genes in GC remains unclear.

To the best of our knowledge, a comprehensive analysis has yet to be applied to clarify the role of SLC30A family genes in GC. Based on the multiple bioinformatics databases, we analyzed the expression and mutation of SLC30A family genes in patients with GC, and evaluated their prognostic value.

## Materials and methods

### Patients and samples

The present study was performed using data obtained from 40 consecutive patients from Cixian and Cixian, a region in Hebei Province with a high rate of epidemiologically and histologically confirmed GC^9,11^. All patients were surgically treated at The Fourth Hospital of Hebei Medical University from January 1, 2017 to December 31, 2018. All patients have received pathological diagnosis of primary GC (Supplementary Table 1).

**Table 1:** Multivariate analysis based on GSE62254.

Factor	Subgroup	β	SE	Wald	RR (95% CI)	*P*
TNM stage	T3	0.686	0.199	11.914	1.986 (1.345–2.933)	0.001
	N2	0.953	0.372	6.578	2.594 (1.252–5.375)	0.010
	N3	1.763	0.382	21.262	5.830 (2.756–12.334)	< 0.001
	M	1.009	0.247	16.712	2.742 (1.691–4.447)	< 0.001
SLC30A2		0.409	0.187	4.762	1.505 (1.042–2.172)	0.029
SLC30A5		− 0.518	0.179	8.357	0.596 (0.419–0.846)	0.004
SLC30A7		− 0.472	0.180	6.863	0.624 (0.439–0.888)	0.009

### RNA isolation and reverse transcription-quantitative polymerase chain reaction (RT-qPCR)

Total RNA was extracted from frozen tumor and corresponding non-tumorous tissues using TRIzol reagent (Invitrogen, Thermo Fisher Scientific, Inc.). After the concentration and purity of the total RNA were determined by ultraviolet absorbance spectroscopy, RNA was reverse transcribed into cDNA using RevertAid First Strand cDNA Synthesis Kit (Thermo Scientific, Lithuania). qRT-PCRs using SuperReal PreMix Plus (SYBR Green) (TianGen, Beijing, China) were performed on ABI7500 Real-Time System (Life Technologies Corp., Foster City, CA, USA). The PCR cycling parameters were as follows: 95 ℃ for 10 min, and 40 cycles of 95 ℃ for 15 s, 60 ℃ for 30 s and 72 ℃ for 30 s. The samples were run in triplicate and the mean value was calculated for each case. The primers for SLC30A family genes are listed in Supplementary Table 2. The human GAPDH gene was employed as an internal control. The relative expression of SLC30A family genes was calculated using the 2^−ΔΔCT^ method according to the previously described protocol^25^.

**Table 2:** The relationship between *SLC30A* family genes and OS in different gender of GC patients (Kaplan–Meier Plotter).

	Gender	Cases	HR (95% CI)	*P*-value
SLC30A1	Male	349	0.54 (0.39–0.76)	0.0004*
	Female	187	0.52 (0.34–0.8)	0.0026*
SLC30A2	Male	349	2 (1.37–2.91)	0.0002*
	Female	187	1.95 (1.11–3.4)	0.0170*
SLC30A3	Male	349	1.67 (1.34–2.07)	2.8e−06*
	Female	187	1.95 (1.36–2.8)	0.0002*
SLC30A4	Male	349	1.52 (1.12–2.06)	0.0065*
	Female	187	1.68 (1.1–2.59)	0.0161*
SLC30A5	Male	349	0.55 (0.4–0.74)	6.6e−05*
	Female	187	0.58 (0.36–0.91)	0.0171*
SLC30A6	Male	349	0.5 (0.37–0.67)	2.7e−06*
	Female	187	0.59 (0.36–0.97)	0.0370*
SLC30A7	Male	349	0.45 (0.33–0.61)	1.3e−07*
	Female	187	0.64 (0.42–0.99)	0.0431*
SLC30A8	Male	349	1.37 (1.02–1.84)	0.0370*
	Female	187	2.49 (1.6–3.87)	2.8e−05*
SLC30A9	Male	349	0.47 (0.38–0.59)	3.7e−11*
	Female	187	0.54 (0.37–0.78)	0.0010*
SLC30A10	Male	349	1.73 (1.32–2.25)	4.8e−05*
	Female	187	1.35 (0.95–1.92)	0.0930*

a: The *P*-value was set at 0.05 and the * indicate that the results are statistically significant.

b: SLC30, The solute carriers’ families 30; OS, overall survival; HR, hazard ratio; CI, confidence interval.

### TCGA database

TCGA is a large repository of high throughput data of human carcinomas, containing over 30 human tumor cohort studies^26^. The expression profiling of SLC30A family genes were retrieved from the TCGA-STAD database. In addition, the clinicopathological parameters of GC were downloaded from TCGA in order to assess the diagnostic value of SLC30A family genes in GC patients using receiver operating characteristic (ROC) curve.

### UALCAN database

UALCAN is a web resource that provides comprehensive cancer transcriptome data (https://ualcan.path.uab.edu/)^27^. The expression level of SLC30A family genes in GC tissues and normal gastric tissues were assessed using the UALCAN database.

### TIMER database analysis

TIMER (https://cistrome.shinyapps.io/timer/) is an a comprehensive and user-friendly online tool to systematically investigate and visualize the correlation between immune infiltrates and a wide spectrum of factors, including gene expression, clinical outcomes and somatic mutations over 10,897 tumors from 32 cancer types^28,29^. The differential expression of SLC30A family genes between tumor and normal tissues could be evaluated using Diff Exp module across all the TCGA database tumors and the results were shown with boxplots.

### cBioportal database

cBioportal is an interactive open-source platform, that provides large scale cancer genomics data sets (https://www.cbioportal.org/)^30,31^. The frequency of SLC30A family gene alterations (amplification, deep deletion, and missense mutations) in GC patients was assessed using the cBioportal for Cancer Genomics database and TCGA.

### Correlation and functional enrichment analysis of SLC30A family Genes

Correlation between the mRNA expression of SLC30A family genes was evaluated using Pearson’s correlation coefficient and Corrplot^32^ package in R software. Gene ontology (GO), including biological process (BP), molecular function (MF) and cellular component (CC), is a commonly used bioinformatics tool that provides comprehensive information on gene function of individual genomic data. The Kyoto Encyclopedia of Genes and Genomes (KEGG), a database was used to assign biological function and utilities of target genes. GO and KEGG enrichment analysis and annotations were performed using the Database for Annotation, Visualization and Integrated Discovery (DAVID) database (https://david.ncifcrf.gov/)^33^, which provides a user-friendly and comprehensive tools for explore the potential biological meaning of what you are interested gene lists. Enrichment results visualization was performed using ClusterProfiler^34^ package in R software with criterion false discovery rate (FDR < 0.05). To understand the connections among the SLC30A family genes, STRING database (https://string-db.org/) was used to construct PPI network^35,36^.

### Kaplan–Meier plotter database

ROC curve analysis was conducted using the pROC^37^ package in R software to explore the sensitivity and specificity of using the SLC30A family genes to distinguish GC patients from healthy individuals. Kaplan–Meier plotter (https://kmplot.com/) is an online database containing microarray gene expression data and survival information extrcated from Gene Expression Omnibus and TCGA database, which contain the gene expression and survival data of 1065 GC patients^38^. 631 GC patients were included in this study (Supplementary Table 3). Patients missing expression values or lacking complete clinical data, including survival time and status, were exclude. To investigate the underlying prognostic value of SLC30A family genes, we evaluated OS, FPS, and PPS using the Kaplan–Meier plotter database based on median expression (high vs. low). Assessments were made using a Kaplan–Meier survival plot with a hazard ratio with 95% confidence intervals and log rank *p*-values. Furthermore, the correlation between mRNA expression of SLC30A family genes and different clinicopathological characteristics such as gender, age, HER2 status, clinical stage, Lauren classification, differentiation, perforation, and treatment method were evaluated using this database. Treatment classification in GC patients was divided into surgery alone, 5 FU-based adjuvant, and other adjuvant treatments. Moreover, we performed multivariate Cox regression analysis to determine if SLC30A family genes could serve as prognostic markers based on GSE62254 cohort.

**Table 3:** The relationship between *SLC30A* family genes and OS in different stages of GC patients (Kaplan–Meier Plotter).

	Stage	Cases	HR (95% CI)	*P*-value
SLC30A1	I	62	0.43 (0.14–1.31)	0.1270
	II	140	0.74 (0.37–1.5)	0.4060
	III	197	0.64 (0.43–0.96)	0.0280*
	IV	140	0.53 (0.34–0.82)	0.0037*
SLC30A2	I	62	3,777,800 (0-lnf)	0.0150*
	II	140	1.55 (0.82–2.9)	0.1710
	III	197	1.69 (1.15–2.49)	0.0067*
	IV	140	2 (1.34–3)	0.0006*
SLC30A3	I	62	2.34 (0.67–8.22)	0.1720
	II	140	1.66 (0.88–3.13)	0.1140
	III	197	1.64 (1.23–2.19)	0.0007*
	IV	140	0.75 (0.51–1.12)	0.1570
SLC30A4	I	62	3.06 (0.93–10.1)	0.0551
	II	140	1.56 (0.81–3.01)	0.1840
	III	197	1.68 (1.14–2.49)	0.0082*
	IV	140	1.6 (1.07–2.38)	0.0210*
SLC30A5	I	62	0.21 (0.07–0.64)	0.0026*
	II	140	1.65 (0.82–3.33)	0.1590
	III	197	0.58 (0.4–0.85)	0.0046*
	IV	140	0.56 (0.37–0.84)	0.0041*
SLC30A6	I	62	0.14 (0.03–0.64)	0.0033*
	II	140	0.55 (0.29–1.05)	0.0650
	III	197	0.57 (0.38–0.84)	0.0042*
	IV	140	0.48 (0.31–0.74)	0.0007*
SLC30A7	I	62	0.19 (0.05–0.7)	0.0053*
	II	140	0.67 (0.34–1.35)	0.2630
	III	197	0.57 (0.39–0.83)	0.0031*
	IV	140	0.63 (0.41–0.98)	0.0390*
SLC30A8	I	62	4.6 (1.53–13.82)	0.0028*
	II	140	1.8 (0.88–3.69)	0.1031
	III	197	1.55 (1.07–2.25)	0.0202*
	IV	140	1.68 (1.1–2.57)	0.0160*
SLC30A9	I	62	0.22 (0.08–0.6)	0.0013*
	II	140	0.58 (0.32–1.08)	0.0850
	III	197	0.53 (0.39–0.72)	4.9e−05*
	IV	140	0.6 (0.41–0.89)	0.0100*
SLC30A10	I	62	2.88 (1.07–7.75)	0.0280*
	II	140	1.7 (0.94–3.07)	0.0780
	III	197	1.54 (1.08–2.18)	0.0150*
	IV	140	0.72 (0.45–1.14)	0.1550

a: The *P*-value was set at 0.05 and the * indicate that the results are statistically significant.

### Single-sample gene set enrichment analysis (ssGSEA)

The infiltration levels of immune cell types were quantified by ssGSEA method using gsva package^39^ in R software. The ssGSEA applies gene signatures expressed by immune cell populations to indivadual cancer samples^40^. The deconvolution approach used in our study including 24 immune cells that are involved in immunity including B cells, DC, iDC, aDC, pDC, Eosinophils, Macrophages, Mast cells, Neutrophils, NK cells, NK CD56dim cells, NKCD56bright cells, T cell, Cytotoxic cells, CD8 T cells, Tgd, T helper cells, Tcm, Tem, Th1, Th2, Tfh, TReg, Th17^41^. And we further conducted the ssGSEA algorithm to explore the relationship between the SLC30A family genes and the infiltration of immune cells.

### Statistical analysis

All statistical analysis was performed using SPSS 21.0 software (SPSS Company, Chicago, Illinois, USA) and R software. And all methods were performed in accordance with the relevant guidelines and regulations. The real-time RT-PCR results were expressed as the mean ± S.D. Student’s test was used to compare the expression means between different groups. *P* < 0.05 indicated a statistically significant difference.

### Ethics statement

This study was approved by the Institutional Human Ethics Committee of Hebei Medical University Fourth Hospital (ID 2018MEC042), and prior informed consent obtained from all the patients. We confirm that all the methods had been carried out in accordance with the relevant guidelines and regulations of the Declaration of Helsinki.

### Consent for publication

All authors have reviewed the manuscript and consented for publication.

## Results

### Relative transcriptional expression of SLC30A family genes in GC patients using the UALCAN database

**Figure 1:**
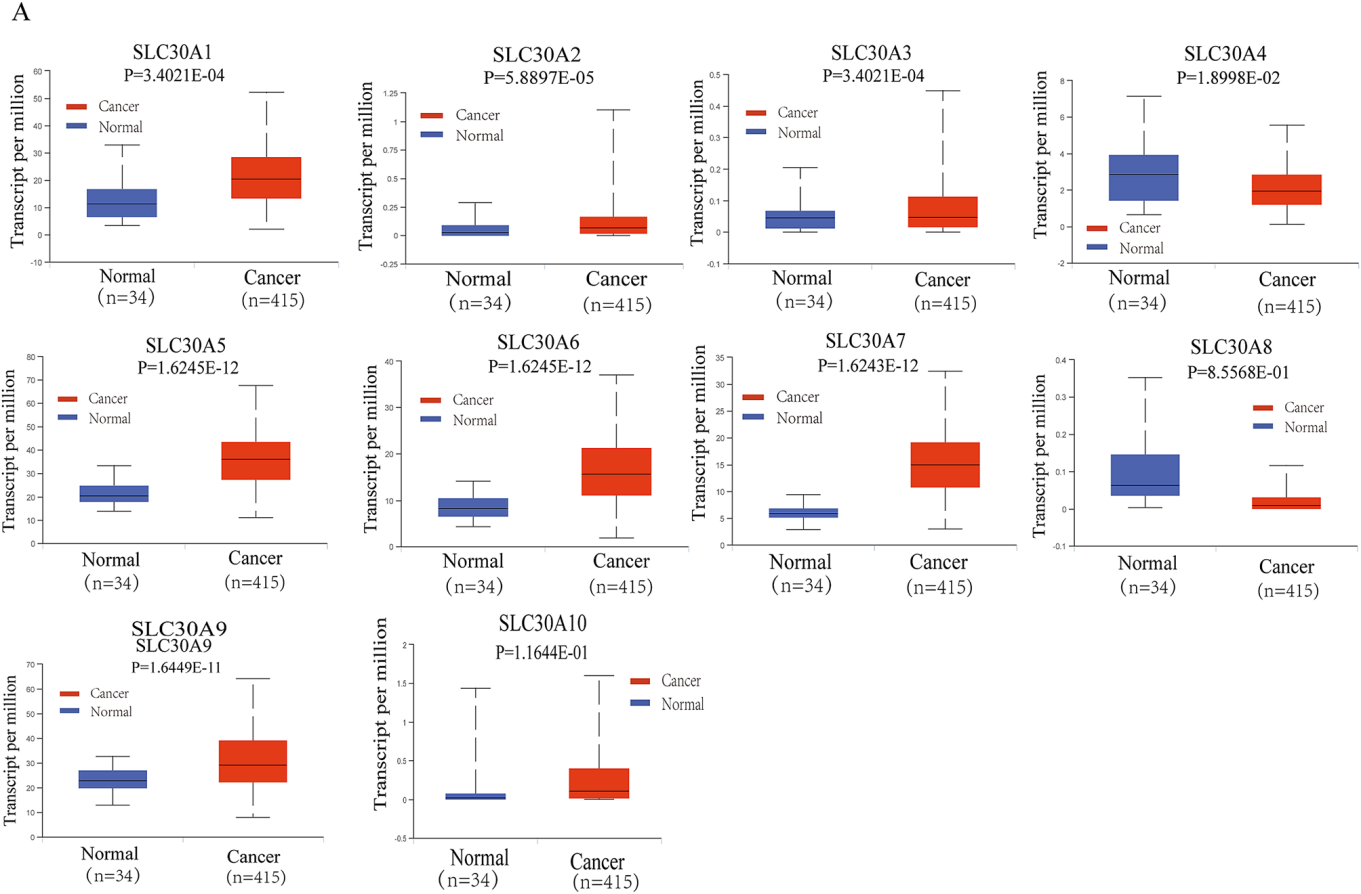

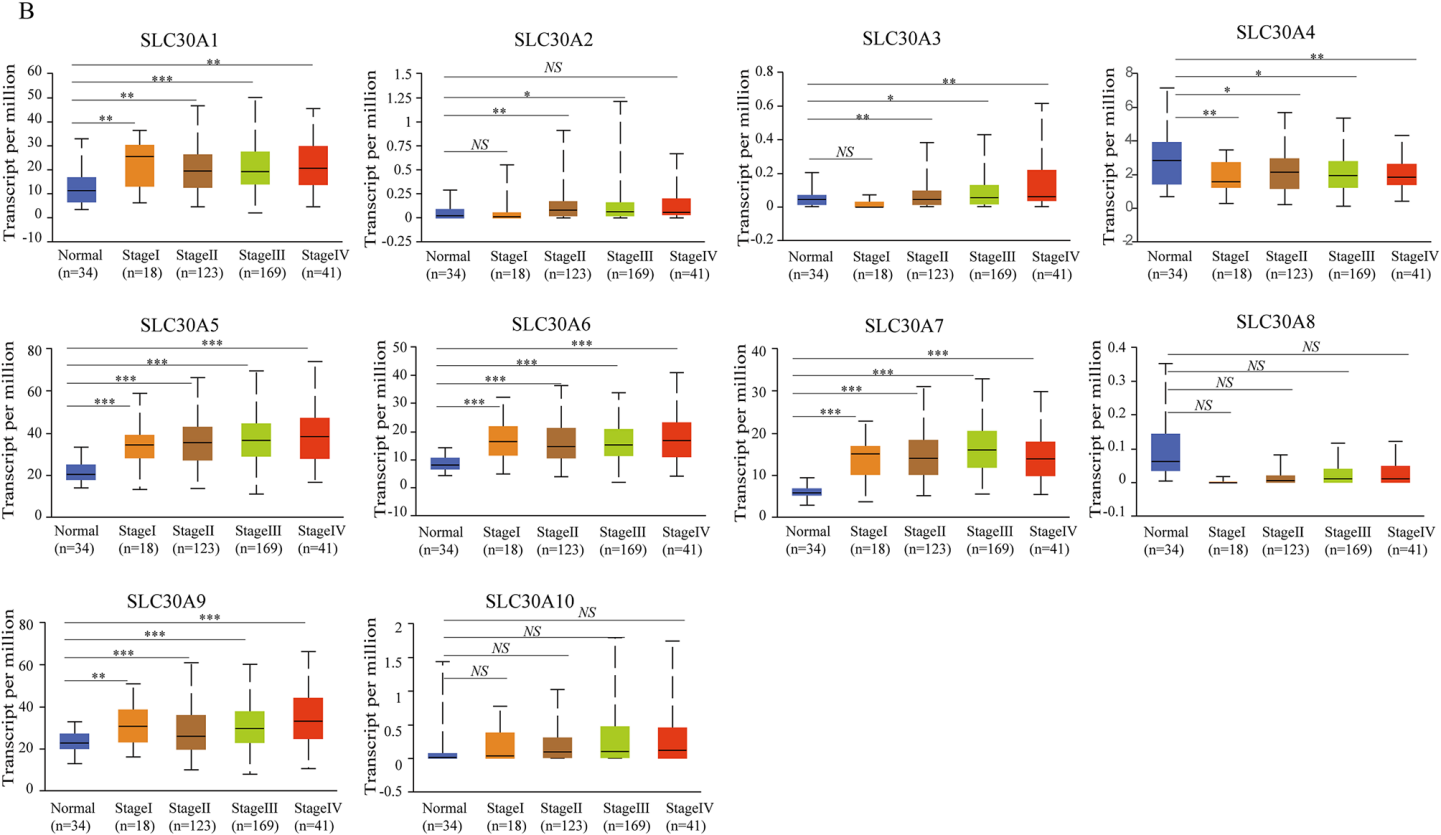

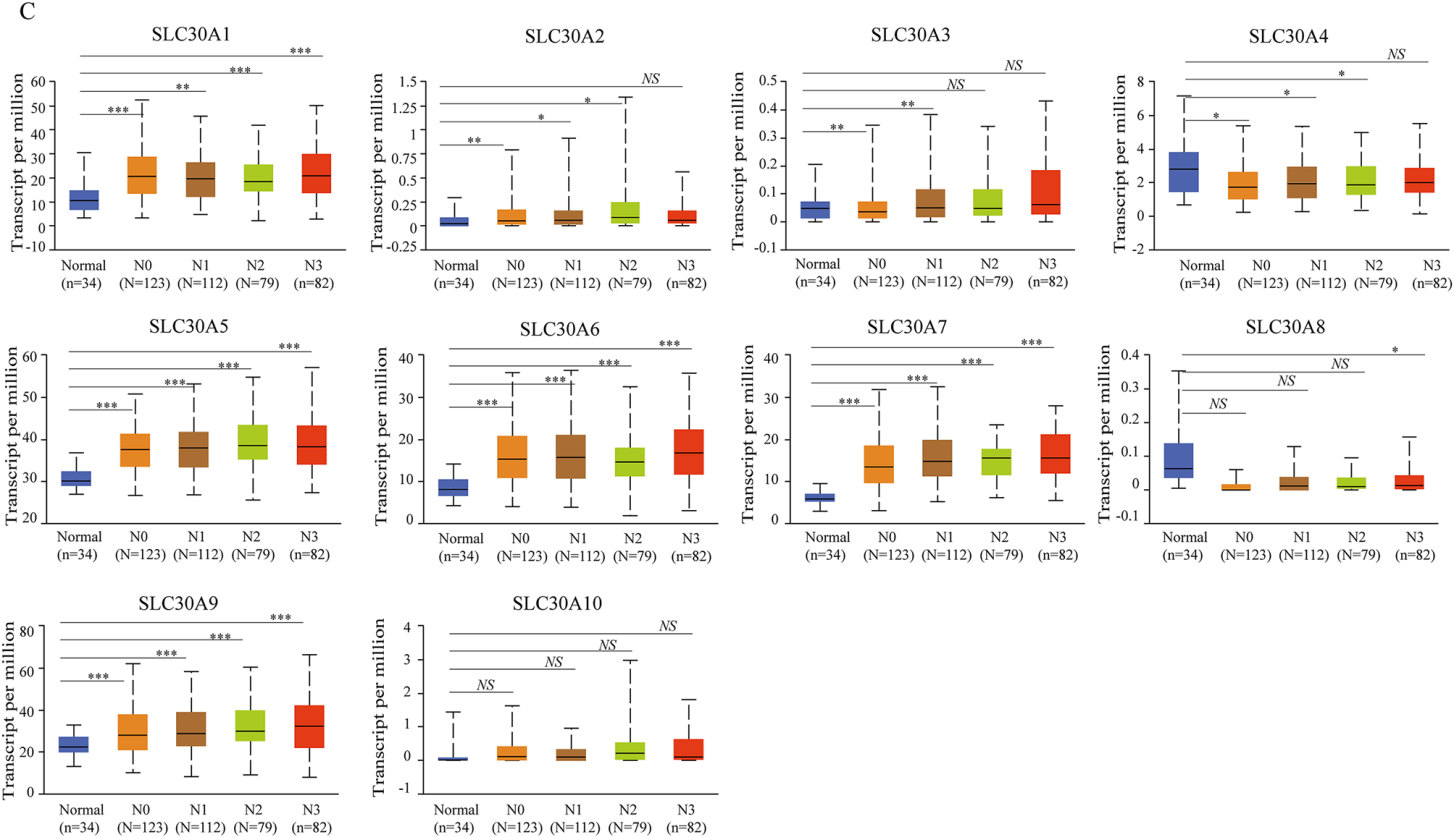

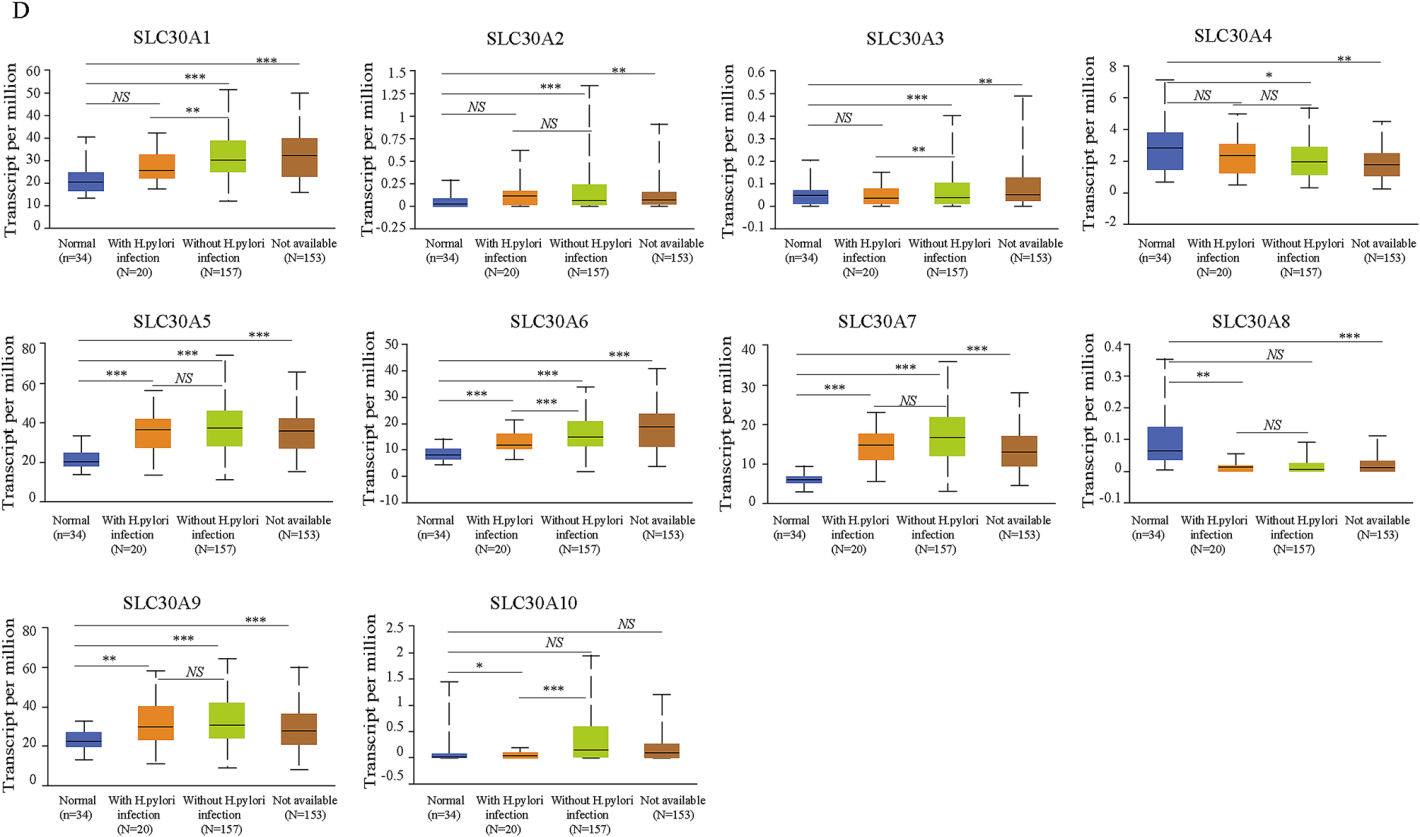

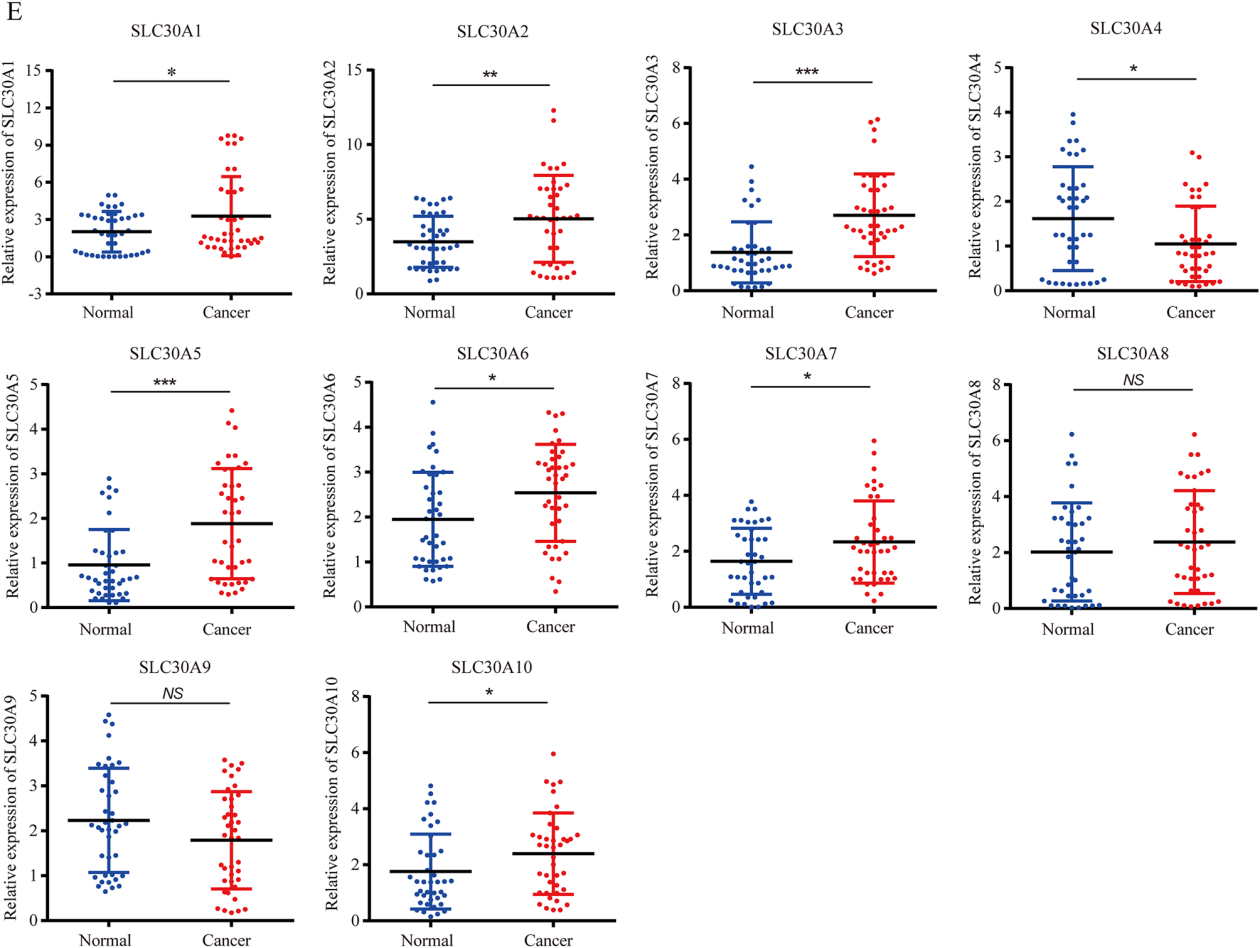
Relative expression and the correlation between SLC30A family genes in patients with GC. (**A**) The expression of SLC30A family genes in GC patients (Ualcan database). The *P*-value was set at 0.05, and most of SLC30A family genes were significantly dysregulated in GC patients. (**B**) Correlation between expression of SLC30A family genes and tumor stages in GC patients (TCGA data). (**C**) Expression of SLC30A family genes in GC based on nodal metastasis status (UALCAN database). N0: No regional lymph node metastasis; N1: metastases in 1 to 3 axillary lymph nodes; N2: metastases in 4 to 9 axillary lymph nodes; N3: metastases in 10 or more axillary lymph nodes. (**D**) expression of SLC30A family genes in GC based on H.pylori infection status (UALCAN database). (**E**) Relative expression of SLC30A family genes validated in 40 patients with GC. The *P*-value was set at 0.05. * indicates *P*-value < 0.05, ** indicates *P*-value < 0.01, *** indicates *P*-value < 0.001, *NS* indicates no statistical significance.

Comparison of the transcriptional expression of SLC30A family genes in gastric tumor tissues and normal tissues indicated that mRNA expression of *SLC30A1-3*, *5–7*, and *9* was significantly upregulated in cancer tissues compared to non-cancerous tissues in GC patients, while *SLC30A4* was downregulated in the former compared to the latter (Fig. 1A and Figure S3). Moreover, assessment of the correlation between SLC30A family genes expression levels and the tumor stages of GC patients indicated that the expression levels of most SLC30A family genes, including *SLC30A1*, *5–7*, and *9*, were significantly and positively associated with tumor stage in GC patients. Nevertheless, *SLC30A8* and 10 expression had no statistical significance (Fig. 1B). We also analyzed the relationship between the expression level of SLC30A family genes and the nodal metastasis status of GC patients. Five genes were positively associated with nodal metastasis for GC patients (*SLC30A1*, *5–7*, and *9*). However, *SLC30A4* was significantly and negatively correlated with nodal metastasis for GC patients (Fig. 1C). The expression level of most SLC30A family genes was significantly associated with the *Helicobacter pylori* infection status of GC patients, but the most significant correlation occurred for *SLC30A5-10* (Fig. 1D). Furthermore, we validated the expression of SLC30A family genes in 40 GC patients. Most of the expression levels of SLC30A family genes were consistent with those of previous studies, but the expression levels of *SLC30A8* and *9* had no significant differences between GC tissues and corresponding non-cancerous tissues (Fig. 1E).

### Diagnostic value of SLC30A family genes for distinguishing GC patients

**Figure 2:**
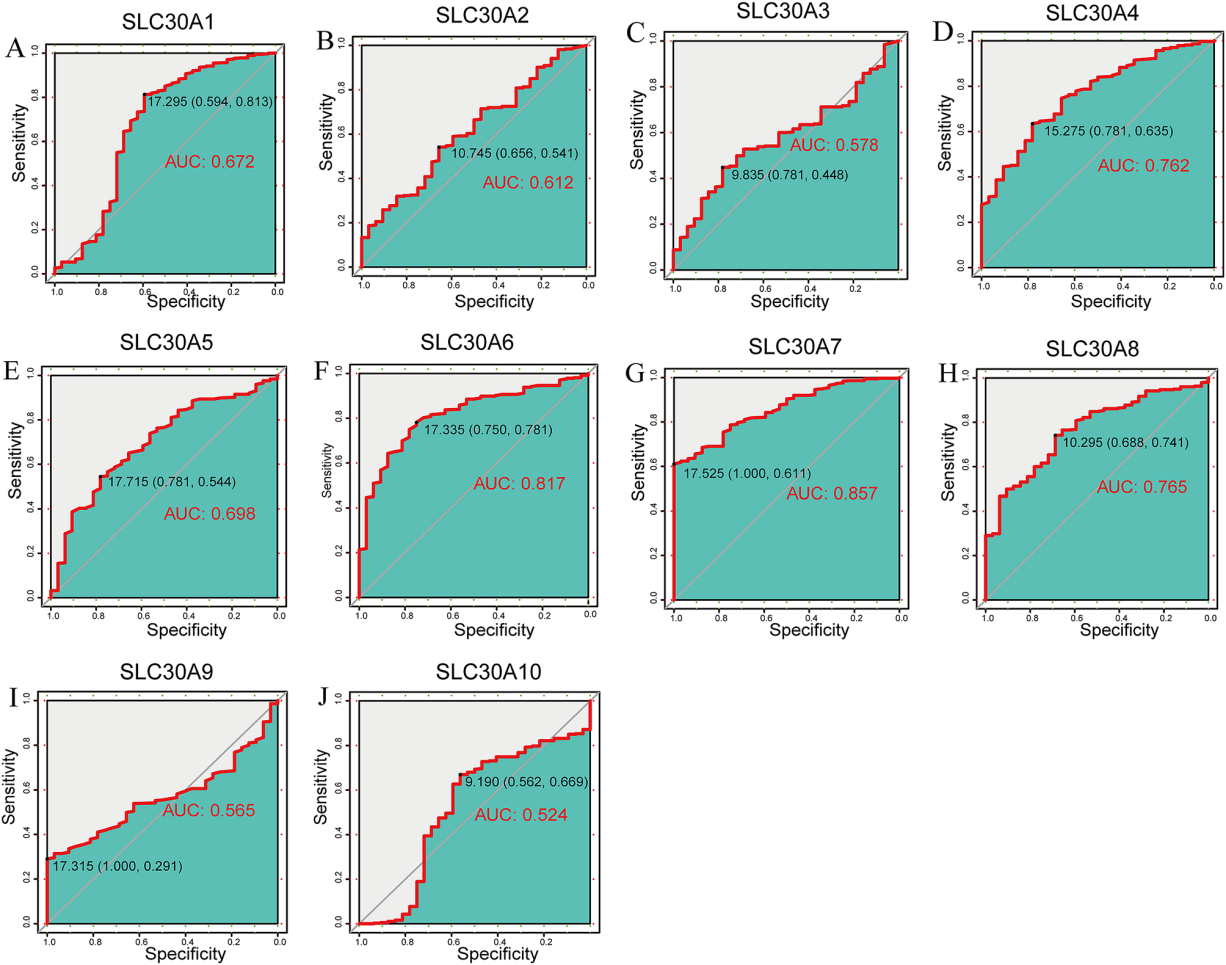
The Receiver operating characteristic (ROC) curve of individual SLC30A family genes. (**A**) SLC30A1; (**B**) SLC30A2; (**C**) SLC30A3; (**D**) SLC30A4; (**E**) SLC30A5; (**F**) SLC30A6; (**G**) SLC30A7; (**H**) SLC30A8; (**I**) SLC30A9; (**J**) SLC30A10.

To assess the diagnostic value of SLC30A family genes in GC patients, we performed a receiver operating characteristic (ROC) curve analysis based on data from the Cancer Genome Atlas (TCGA) database. ROC analysis of the predictive efficiency of SLC30A family genes suggested that most of these genes had high diagnostic value for distinguishing GC patients from healthy individuals, including SLC30A1 (0.672), SLC30A2 (0.612), SLC30A4 (0.762), SLC30A5 (0.698), SLC30A6 (0.817), SLC30A7 (0.857), and SLC30A8 (0.765). SLC30A3 (0.578), SLC30A9 (0.565), and SLC30A10 (0.524) had moderate value for distinguishing GC patients (Fig. 2).

### Prognostic value of SLC30A family genes in GC patients

**Figure 3:**
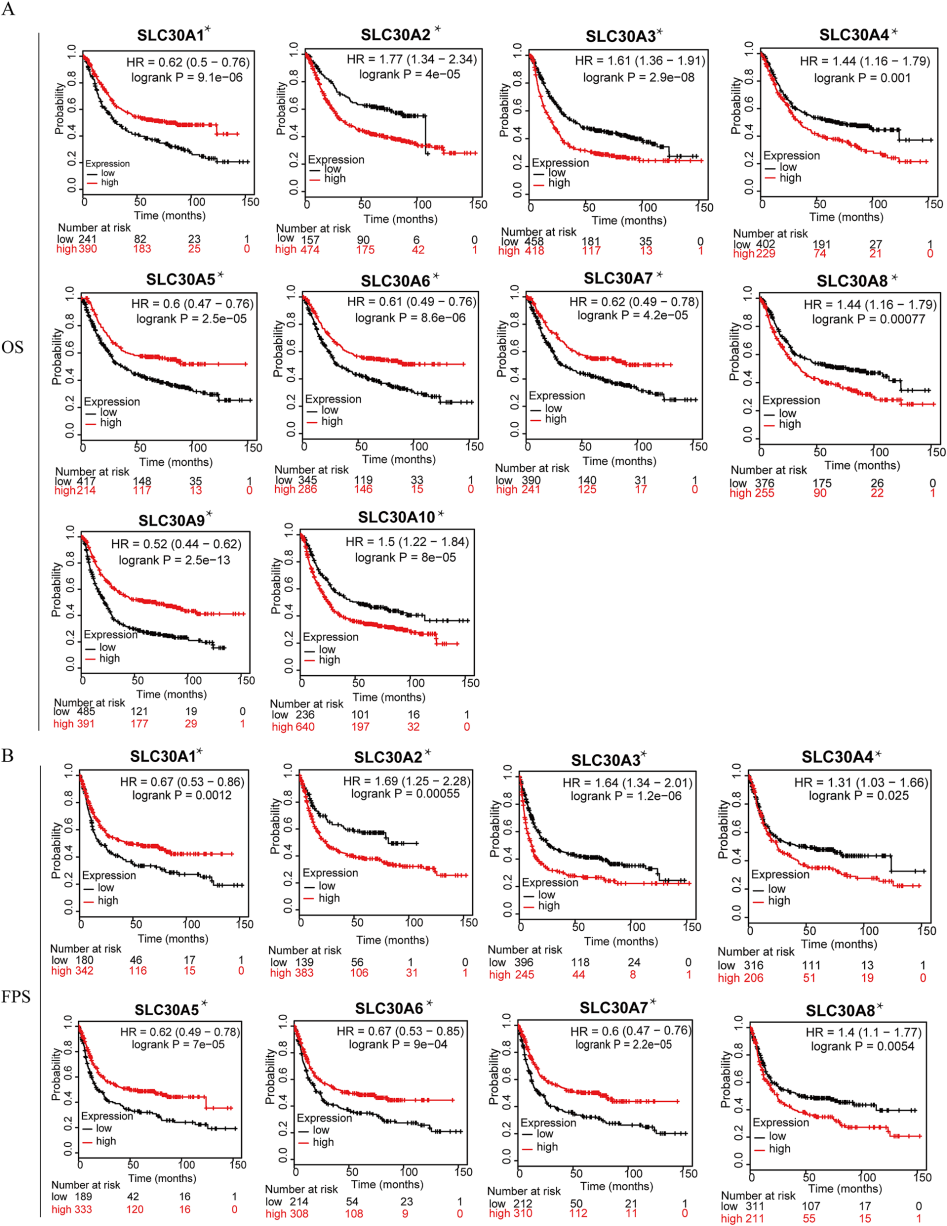

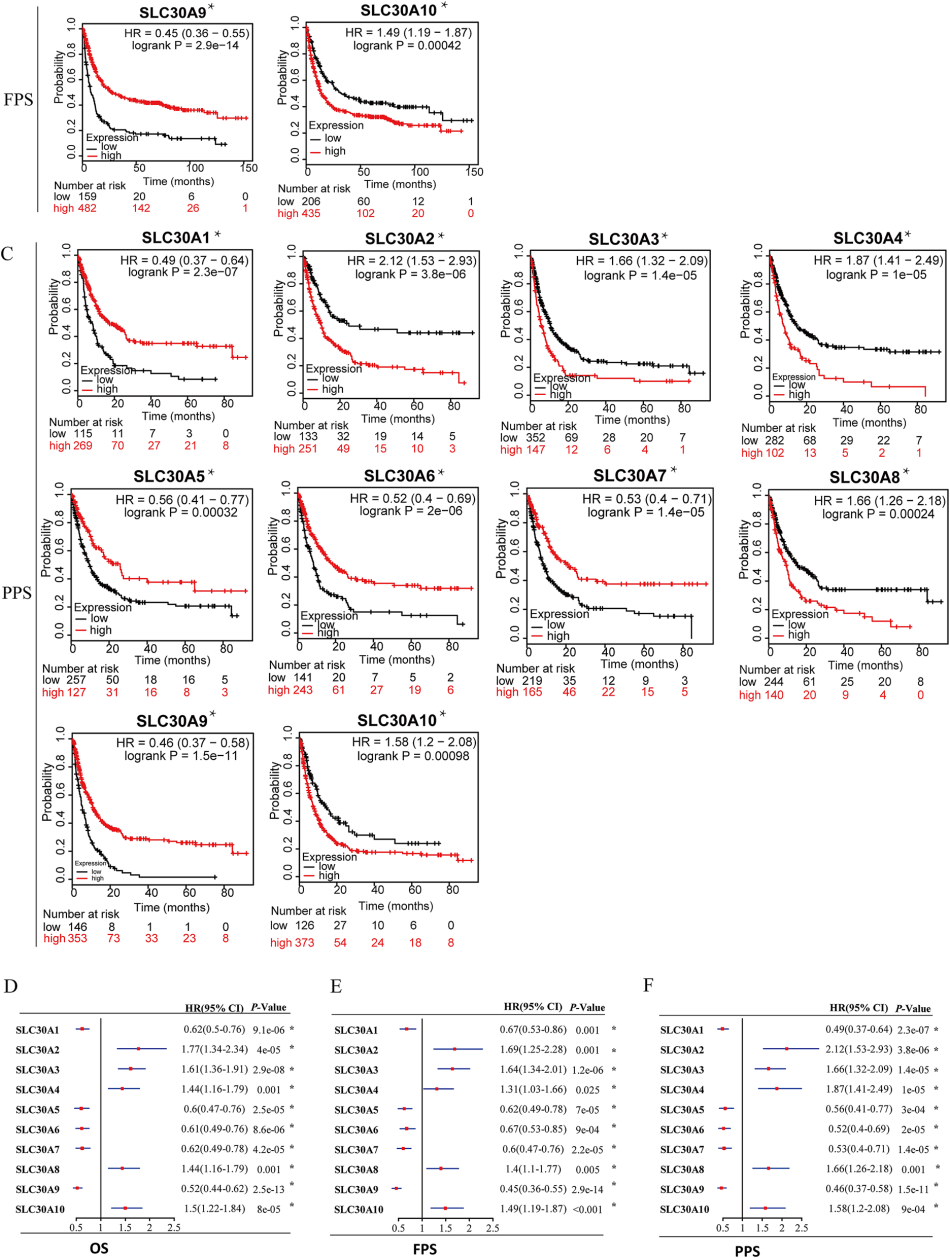
Prognostic value of SLC30A family genes in GC patients. (**A-C**) The correlation between expression level of SLC30A family genes and OS, FPS, and PPS in GC patients (Kaplan–Meier plotter database). (**D-F**) Forest plot of OS, FPS, PPS and mRNA expression of SLC30A family genes in GC patients. Logrank *P* was set at 0.05. OS: overall survival. FPS: First Progression Survival; PPS: Post Progression Survival.

As shown in Fig. 3, all genes were significantly correlated with prognosis in GC patients. Five genes showed a significantly better OS in GC patients when upregulated, *(SLC30A1*: HR 0.62 [95% CI 0.5–0.76], *P* = 9.1e−06; *SLC30A5*: HR 0.6 [95% CI 0.47–0.76], *P* = 2.5e−05; *SLC30A6*: HR 0.61 [95% CI 0.49–0.79], *P* = 8.6e−06; *SLC30A7*: HR 0.62 [95% CI 0.49–0.78], *P* = 4.2e−05; and *SLC30A9*: HR 0.52, [95% CI 0.44–0.62], *P* = 2.5e−13). Five genes showed a negative correlaion between high expression and significant positive overall survival in GC patients, (*SLC3A2*: HR 1.77 [95% CI 1.34–2.34], *P* = 4e−05; *SLC30A3*: HR 1.61 [95% CI 1.36–1.91], *P* = 0.9e−08; *SLC30A4*: HR 1.44 [95% CI 1.16–1.79], *P* = 0.0010; *SLC30A8*: HR 1.44 [95% CI 1.16–1.79], *P* = 0.0008; and *SLC30A10*: HR 1.5 [95% CI 1.22–1.84], *P* = 8e−05). Moreover, multivariate Cox regression analysis indicated that SLC30A2, 5 and 7 could serve as OS markers independent of clinicopathological parameters (Table 1).

Using a forest plot to investigate the potential prognostic value of SLC30A family genes, to reveal the correlation between OS, FPS, PPS, and mRNA expression of SLC30A family genes in GC patients (Fig. 3D–F). The results showed that the high expression of five genes, (*SLC30A1*, *5*–*7*, and *9*), had a positively significant correlation with improved FPS, and PPS. In contrast, upregulated *SLC30A2*-4, 8, and 10 expression was negatively correlated with favorable FPS, and PPS.

### Association of SLC30A family genes prognostic values in GC patients with different clinicopathological features

Investigation of the correlation between clinicopathological features such as gender, clinical stage, Lauren classification, differentiation, HER2 status, treatment types, and perforation and mRNA expression level of SLC30A family genes showed that all SLC30A family gene expression was significantly correlated with gender in GC patients (Table 2). Five genes were promising positive prognostic factors in both male and female patients, including *SLC30A1*, *5–7*, and *9*. Nevertheless, *SLC30A2-4*, *8*, and *10* were significantly correlated with poor prognosis in both male and female patients. Upregulated expression of *SLC30A1*, *5–7*, and *9* predicted a favorable prognosis in GC patients with stage III/IV, I/III/IV, I/III/IV, I/III/IV, and I/III/IV, respectively (Table 3). High expression of *SLC30A2-4*, *8*, and *10* was significantly associated with an unfavorable prognosis in stage I/III/IV, III, III/IV, I/III/IV, and I/III GC patients, respectively.

*SLC30A1*, *3*, *5–7*, and *9* were promising favorable prognostic factors in both intestinal and diffuse type GC patients, and high *SLC30A5* expression was also significantly correlated with mixed type patients (Table 4). Besides, *SLC30A2* and *SLC30A8* predicted poorer prognosis in both intestinal and diffuse type patients and high expression of *SLC30A3* and *SLC30A10* correlated with poor prognosis in intestinal, mixed type GC patients, respectively. High expression of *SLC30A2*, *4*, and *9* were correlated with the improved prognosis in poorly differentiation GC patients (Table 5). Nevertheless, SLC30A1 and 6 were significantly associated with poor OS in moderately differentiation GC patients. Analysis of HER2 status and expression of SLC30A family genes revealed that upregulated expression of *SLC30A1*, *5*–*6*, and *9* predicted favorable OS in both HER2-positive and HER2-negative patients, while *SLC30A2-3*, *8*, and *10* were associated with a worse prognosis. High expression of *SLC30A4* and *7* were significantly associated with unfavorable OS in HER2-negative and improved prognosis in HER2-positive patients, respectively. In this study, treatments in GC patients divided into surgery alone, 5 FU based adjuvant and other treatment (Table 6).

**Table 4:** The relationship between *SLC30A* family genes and OS in different Lauren classification of GC patients (Kaplan–Meier Plotter).

	Lauren classification	Cases	HR (95% CI)	*P*-value
SLC30A1	Intestinal	269	0.59 (0.4–0.87)	0.0065*
	Diffuse	240	0.66 (0.47–0.94)	0.0190*
	Mixed	29	0.36 (0.08–1.63)	0.1670
SLC30A2	Intestinal	269	1.89 (1.21–2.94)	0.0043*
	Diffuse	240	1.97 (1.26–3.06)	0.0023*
	Mixed	29	2.18 (0.66–7.21)	0.1890
SLC30A3	Intestinal	269	1.6 (1.12–2.27)	0.0086*
	Diffuse	240	1.28 (0.88–1.85)	0.1900
	Mixed	29	0.38 (0.14–1.06)	0.0560
SLC30A4	Intestinal	269	1.73 (1.2–2.49)	0.0028*
	Diffuse	240	1.34 (0.95–1.89)	0.0980
	Mixed	29	2.95 (0.97–8.97)	0.0460*
SLC30A5	Intestinal	269	0.44 (0.31–0.64)	7.7e−06*
	Diffuse	240	0.51 (0.34–0.76)	0.0009*
	Mixed	29	0.56 (0.41–0.79)	0.0079*
SLC30A6	Intestinal	269	0.54 (0.37–0.78)	8e−04*
	Diffuse	240	0.62 (0.43–0.88)	0.0070*
	Mixed	29	0.53 (0.18–1.58)	0.245
SLC30A7	Intestinal	269	0.53 (0.36–0.78)	0.0010*
	Diffuse	240	0.54 (0.38–0.76)	0.0003*
	Mixed	29	1.98 (0.52–7.55)	0.3100
SLC30A8	Intestinal	269	1.51 (1.03–2.2)	0.0330*
	Diffuse	240	1.75 (1.24–2.46)	0.0012*
	Mixed	29	2.78 (0.92–8.41)	0.0590
SLC30A9	Intestinal	269	0.42 (0.31–0.58)	4.2e−08*
	Diffuse	240	0.46 (0.3–0.71)	0.0004*
	Mixed	29	0.5 (0.17–1.44)	0.1900
SLC30A10	Intestinal	269	1.41 (0.95–2.11)	0.0880
	Diffuse	240	0.7 (0.46–1.05)	0.0816
	Mixed	29	3.57 (1.23–10.35)	0.0120*

a: The *P*-value was set at 0.05 and the * indicate that the results are statistically significant.

**Table 5:** The relationship between *SLC30A* family genes and OS in different differentiation of GC patients (Kaplan–Meier Plotter).

	Differentiation	Cases	HR (95% CI)	*P*-value
SLC30A1	Poorly	121	1.55 (0.93–2.6)	0.0920
	Moderately	67	2.41 (1.22–4.77)	0.0094*
	Well	5	–	–
SLC30A2	Poorly	121	0.58 (0.35–0.95)	0.0290*
	Moderately	67	1.73 (0.79–3.78)	0.1680
SLC30A3	Poorly	121	0.78 (0.52–1.17)	0.2240
	Moderately	67	1.35 (0.7–2.6)	0.3770
SLC30A4	Poorly	121	0.59 (0.36–0.96)	0.0320*
	Moderately	67	1.66 (0.85–3.22)	0.1334
SLC30A5	Poorly	121	1.22 (0.75–1.98)	0.4230
	Moderately	67	0.66 (0.34–1.28)	0.2150
SLC30A6	Poorly	121	1.54 (0.92–2.55)	0.0950
	Moderately	67	2.03 (1.05–3.95)	0.0330*
SLC30A7	Poorly	121	1.52 (0.92–2.53)	0.1020
	Moderately	67	1.56 (0.8–3.02)	0.1850
SLC30A8	Poorly	121	1.95 (1.11–3.43)	0.0180*
	Moderately	67	0.66 (0.33–1.31)	0.2340
SLC30A9	Poorly	121	0.62 (0.41–0.92)	0.0180*
	Moderately	67	0.6 (0.3–1.16)	0.120
SLC30A10	Poorly	121	0.77 (0.51–1.16)	0.214
	Moderately	67	0.75 (0.37–1.52)	0.4260

a: The *P*-value was set at 0.05 and the * indicate that the results are statistically significant.

**Table 6:** The relationship between *SLC30A* family genes and OS in different HER2 status of GC patients (Kaplan–Meier Plotter).

	HER2	Cases	HR (95% CI)	*P*-value
SLC30A1	Positive	202	0.61 (0.41–0.93)	0.0200*
	Negative	429	0.66 (0.52–0.82)	0.0003*
SLC30A2	Positive	202	1.46 (1–2.14)	0.0470*
	Negative	429	1.67 (1.27–2.18)	0.0002*
SLC30A3	Positive	202	1.6 (1.23–2.08)	0.0004*
	Negative	429	1.58 (1.25–1.98)	8.2e−05*
SLC30A4	Positive	202	1.3 (0.86–1.95)	0.2120
	Negative	429	1.65 (1.26–2.16)	0.0002*
SLC30A5	Positive	202	0.6 (0.42–0.88)	0.0077*
	Negative	429	0.59 (0.46–0.78)	0.0001*
SLC30A6	Positive	202	0.56 (0.36–0.88)	0.0120*
	Negative	429	0.53 (0.41–0.69)	1.9e−06*
SLC30A7	Positive	202	0.69 (0.45–0.76)	4.8e−05*
	Negative	429	0.58 (0.45–1.05)	0.0830
SLC30A8	Positive	202	1.52 (1.05–2.2)	0.0270*
	Negative	429	1.54 (1.16–2.05)	0.0026*
SLC30A9	Positive	202	0.56 (0.42–0.73)	2.2e−05*
	Negative	429	0.48 (0.38–0.61)	6.2e−10*
SLC30A10	Positive	202	1.66 (1.2–2.29)	0.0018*
	Negative	429	1.29 (1.01–1.65)	0.0430*

a: The *P*-value was set at 0.05 and the * indicate that the results are statistically significant.

b: HER2, human epidermal growth factor receptor 2.

**Figure 4:**
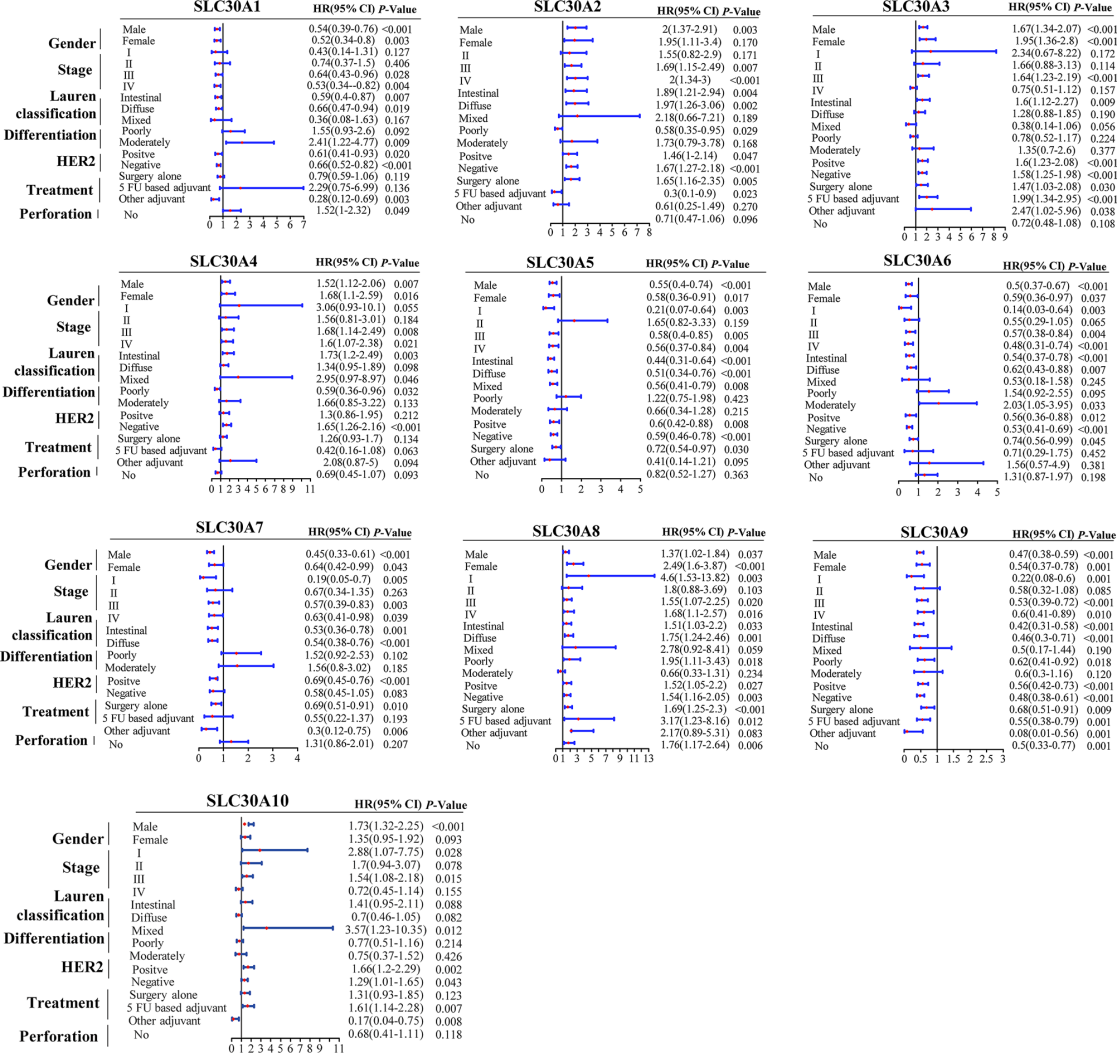
Forest plot of individuals expression level of SLC30A family genes with OS in different clinicopathological features patients with GC (The *P*-value was set at 0.05).

*SLC30A6-7* and *9* were strongly related to favorable OS in GC patients based on a surgery only treatment. *SLC30A1* and *9–10* were positively associated with other adjuvant treatments, while high expression of *SLC30A2* predicted better prognosis in 5 fluorouracil (FU)- based adjuvant treatment. Nevertheless, overexpression of *SLC30A2-3* and *8* were correlated with poor prognosis in patients that received surgery alone. SLC30A3, 8, and 10 were strongly negatively associated with OS in patients that received 5-FU based adjuvant treatment (Table 7). Furthermore, analysis of the correlation between mRNA expression of SLC30A family genes and prognosis in patients with no perforation showed that *SLC30A9* was a favorable factor in patients without perforation, while overexpression of SLC30A*1* and *8* were significantly associated with poor prognosis (Table 8). Taken together, all SLC30A family genes were strongly correlated with clinical characteristics including gender, clinical stage, Lauren classification, differentiation, HER2 status, perforation, and treatment method (Fig. 4).

**Table 7:** The relationship between *SLC30A* family genes and OS in treatment methods of GC patients (Kaplan–Meier Plotter).

	Treatment	Cases	HR (95% CI)	*P*-value
SLC30A1	Surgery alone	380	0.79 (0.59–1.06)	0.1190
	5 FU based adjuvant	34	2.29 (0.75–6.99)	0.1360
	Other adjuvant	76	0.28 (0.12–0.69)	0.0030*
SLC30A2	Surgery alone	380	1.65 (1.16–2.35)	0.0051*
	5 FU based adjuvant	34	0.3 (0.1–0.9)	0.0230*
	Other adjuvant	76	0.61 (0.25–1.49)	0.2700
SLC30A3	Surgery alone	380	1.47 (1.03–2.08)	0.0300*
	5 FU based adjuvant	34	1.99 (1.34–2.95)	0.0005*
	Other adjuvant	76	2.47 (1.02–5.96)	0.0380*
SLC30A4	Surgery alone	380	1.26 (0.93–1.7)	0.1340
	5 FU based adjuvant	34	0.42 (0.16–1.08)	0.0630
	Other adjuvant	76	2.08 (0.87–5)	0.0940
SLC30A5	Surgery alone	380	0.72 (0.54–0.97)	0.0300*
	5 FU based adjuvant	34	2.22 (0.51–9.66)	0.2750
	Other adjuvant	76	0.41 (0.14–1.21)	0.0950
SLC30A6	Surgery alone	380	0.74 (0.56–0.99)	0.0450*
	5 FU based adjuvant	34	0.71 (0.29–1.75)	0.4520
	Other adjuvant	76	1.56 (0.57–4.3)	0.3810
SLC30A7	Surgery alone	380	0.69 (0.51–0.91)	0.0098*
	5 FU based adjuvant	34	0.55 (0.22–1.37)	0.1930
	Other adjuvant	76	0.3 (0.12–0.75)	0.0060*
SLC30A8	Surgery alone	380	1.69 (1.25–2.3)	0.0006*
	5 FU based adjuvant	34	3.17 (1.23–8.16)	0.0120*
	Other adjuvant	76	2.17 (0.89–5.31)	0.0830
SLC30A9	Surgery alone	380	0.68 (0.51–0.91)	0.0085*
	5 FU based adjuvant	34	0.55 (0.38–0.79)	0.0010*
	Other adjuvant	76	0.08 (0.01–0.56)	0.0010*
SLC30A10	Surgery alone	380	1.31 (0.93–1.85)	0.1234
	5 FU based adjuvant	34	1.61 (1.14–2.28)	0.0067*
	Other adjuvant	76	0.17 (0.04–0.75)	0.0081*

a: The *P*-value was set at 0.05 and the * indicate that the results are statistically significant.

b: FU, fluorouracil.

**Table 8:** The relationship between *SLC30A* family genes and OS of GC patients with no perforation (Kaplan–Meier Plotter).

	Perforation	Cases	HR (95% CI)	*P*-value
SLC30A1	No	169	1.52 (1–2.32)	0.0490*
SLC30A2	No	169	0.71 (0.47–1.06)	0.0960
SLC30A3	No	169	0.72 (0.48–1.08)	0.1080
SLC30A4	No	169	0.69 (0.45–1.07)	0.0930
SLC30A5	No	169	0.82 (0.52–1.27)	0.3630
SLC30A6	No	169	1.31 (0.87–1.97)	0.1980
SLC30A7	No	169	1.31 (0.86–2.01)	0.2070
SLC30A8	No	169	1.76 (1.17–2.64)	0.0060*
SLC30A9	No	169	0.5 (0.33–0.77)	0.0012*
SLC30A10	No	169	0.68 (0.41–1.11)	0.1181

a: The *P*-value was set at 0.05 and the * indicate that the results are statistically significant.

### Genetic alteration differences of SLC30A family genes in GC patients

**Figure 5:**
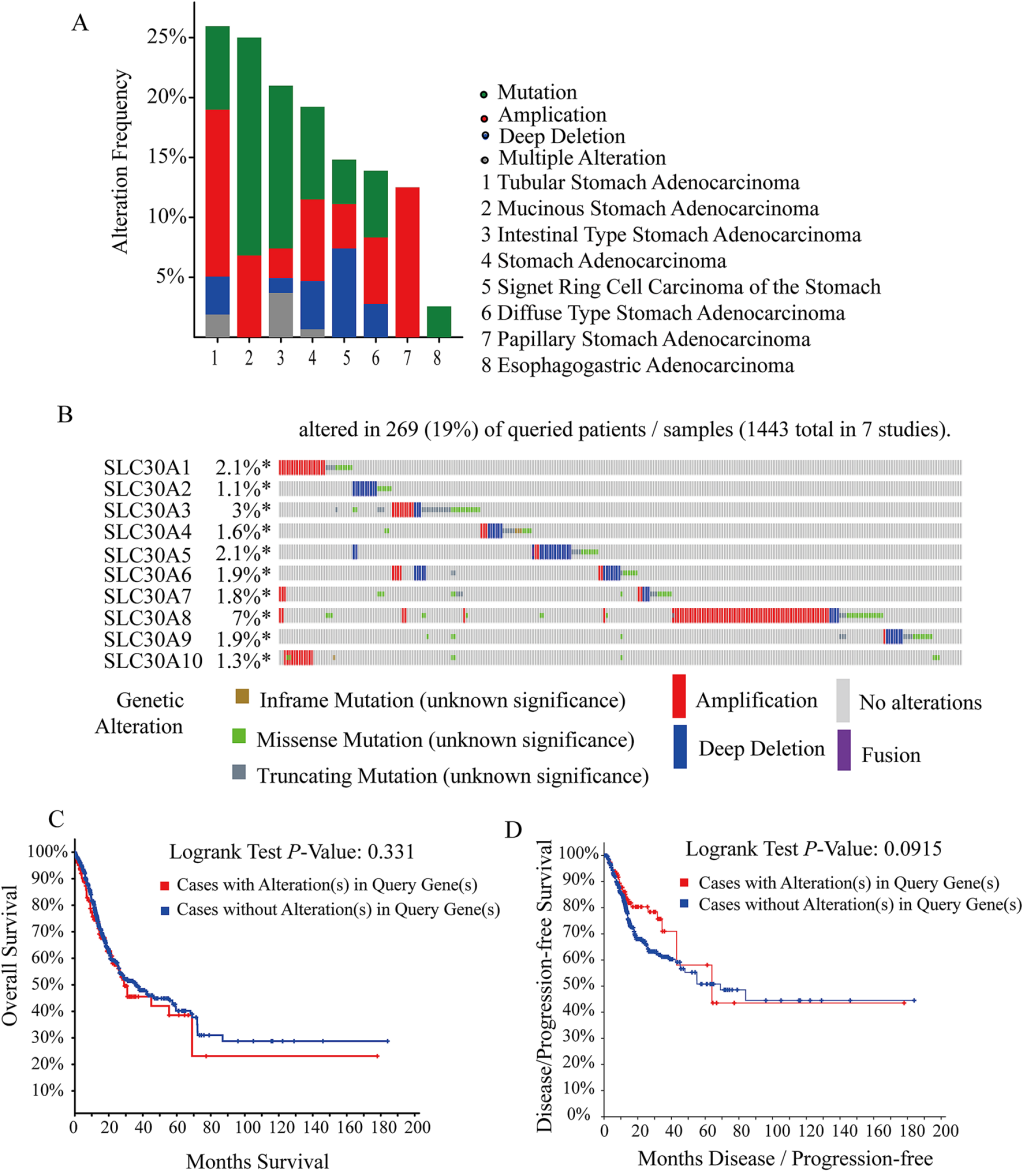
Oncoprint and alteration differences of SLC30A family genes in gastric cancer (cBioportal database). (**A**) summary of alteration in SLC30Afamily genes. (**B**) The visual summary Oncoprint based on a query of the SLC30A family genes. (**C**) Kaplan–Meier plots comparing Overall Survival (OS) in cases with and without SLC30A family genes alterations. (**D**) Kaplan–Meier plots comparing Disease-free Survival (DFS) in cases with and without SLC30A family genes alterations.

To explore the roles of SLC30A family genes in GC patients, genetic alteration of 10 genes was performed using the cBioportal database. A total of 1443 patients from seven GC studies were analyzed. As results showed that mRNA mutation, amplification and deep deletion were the most important factors for alteration in different GC subtypes, including tubular stomach adenocarcinoma, mucinous stomach adenocarcinoma, intestinal type stomach adenocarcinoma, stomach adenocarcinoma, signet ring cell carcinoma of the stomach, diffuse type stomach adenocarcinoma, papillary stomach adenocarcinoma and esophagogastric adenocarcinoma (Fig. 5A). As Fig. 5B shows that SLC30A family genes were altered in 269 samples of 1443 GC patients (19%). The genetic alteration percentages of SLC30A family genes for GC varied from 1.1% to 7% for individual genes (*SLC30A1*, 2.1%; *SLC30A2*, 1.1%; *SLC30A3*, 3%; *SLC30A4*, 1.6%; *SLC30A5*, 2.1%; *SLC30A6*, 1.9%; *SLC30A7*, 1.8%; *SLC30A8*, 7%; *SLC30A9*, 1.9%; *SLC30A10*, 1.3%). The results of Kaplan–Meier plotter and log-rank test showed no significantly statistical difference in overall survival (OS) and disease-free survival (DFS) in cases with and without SLC30A family genes alterations (*P*-value was 0.331 and 0.0915, respectively. Figure 5C,D).

### Correlation and functional enrichment analysis of SLC30A family genes

**Figure 6:**
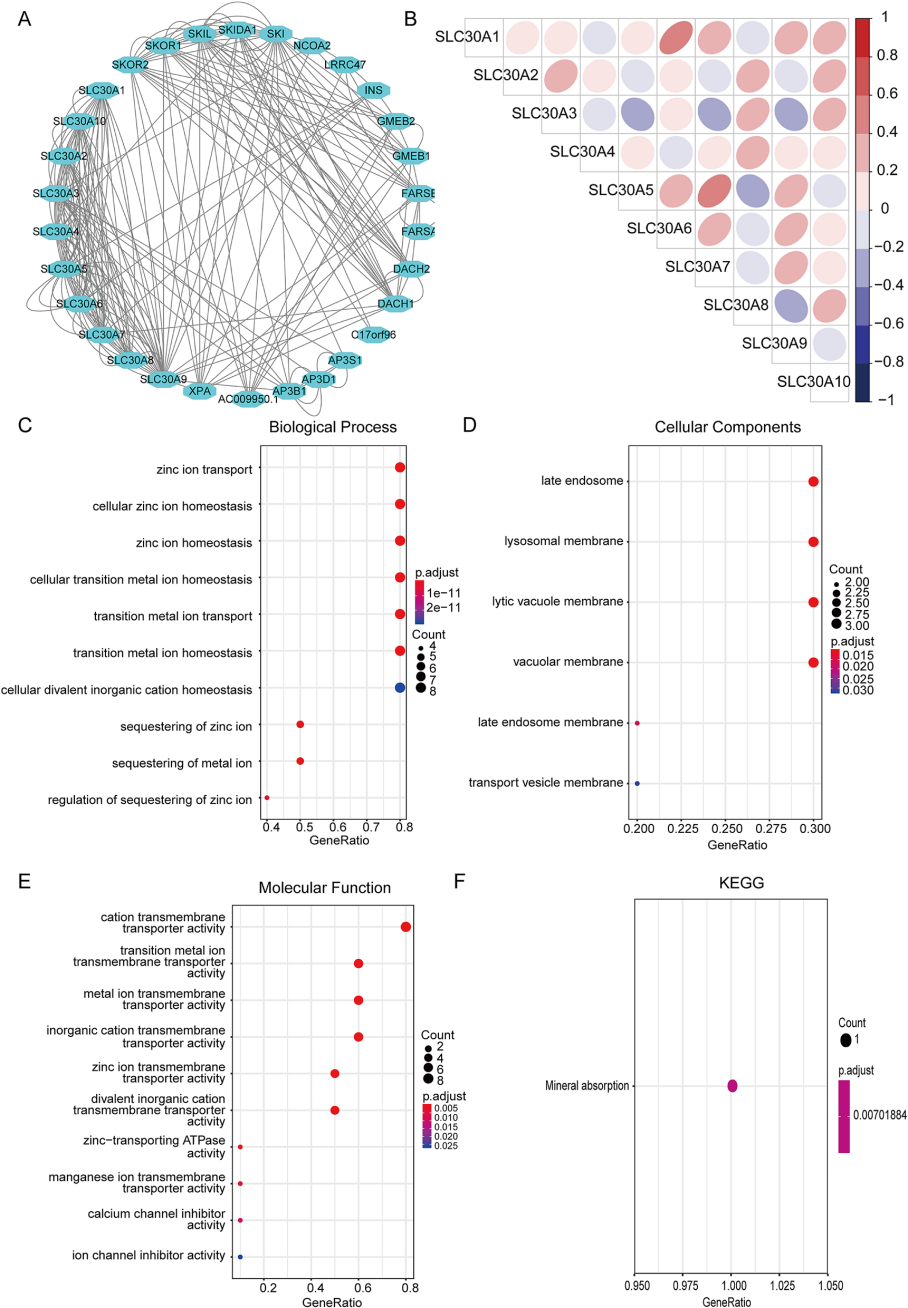
Correlation and functional enrichment analysis of SLC30A family genes. (**A**) Protein–protein interaction network analysis using STRING database. (**B**) Pearson correlation analysis of individual among SLC30A family genes. (**C**) Biological process analysis; (**D**) cellular components; (**E**) molecular function. (**F**) Kyoto Encyclopedia of Genes and Genomes (KEGG) analysis. All of terms colored by adjusted *P*-value and the size of points represent number of genes.

To further reveal the potential functional mechanisms in GC patients, we constructed the correlation between the expression of SLC30A family genes, protein–protein interaction (PPI) network, gene ontology (GO) term analysis, and Kyoto Encyclopedia of Genes and Genomes (KEGG) enrichment analysis (Fig. 6). The individual mRNA expressions of SLC30A family genes in GC patients were weakly correlated (Fig. 6B). The PPI network showed that 30 genes including XPA, FARSB, DACH1, and DACH2 participated in PPI networks through multiple pathways, physical interactions, genetic interactions, shared protein domains and co-expression (Fig. 6A). SLC30A family genes and their neighboring genes were mainly involved in the zinc transport, cellular zinc ion homeostasis, zinc ion homeostasis, cellular transition metal ion homeostasis, and transition metal ion transport, which are mineral transport related biological processes and mineral absorption pathways analyzed by GO term analysis and KEGG pathway enrichment analysis (Fig. 6C–F).

### Immune infiltrates in correlation with SLC30A family genes in GC

**Figure 7:**
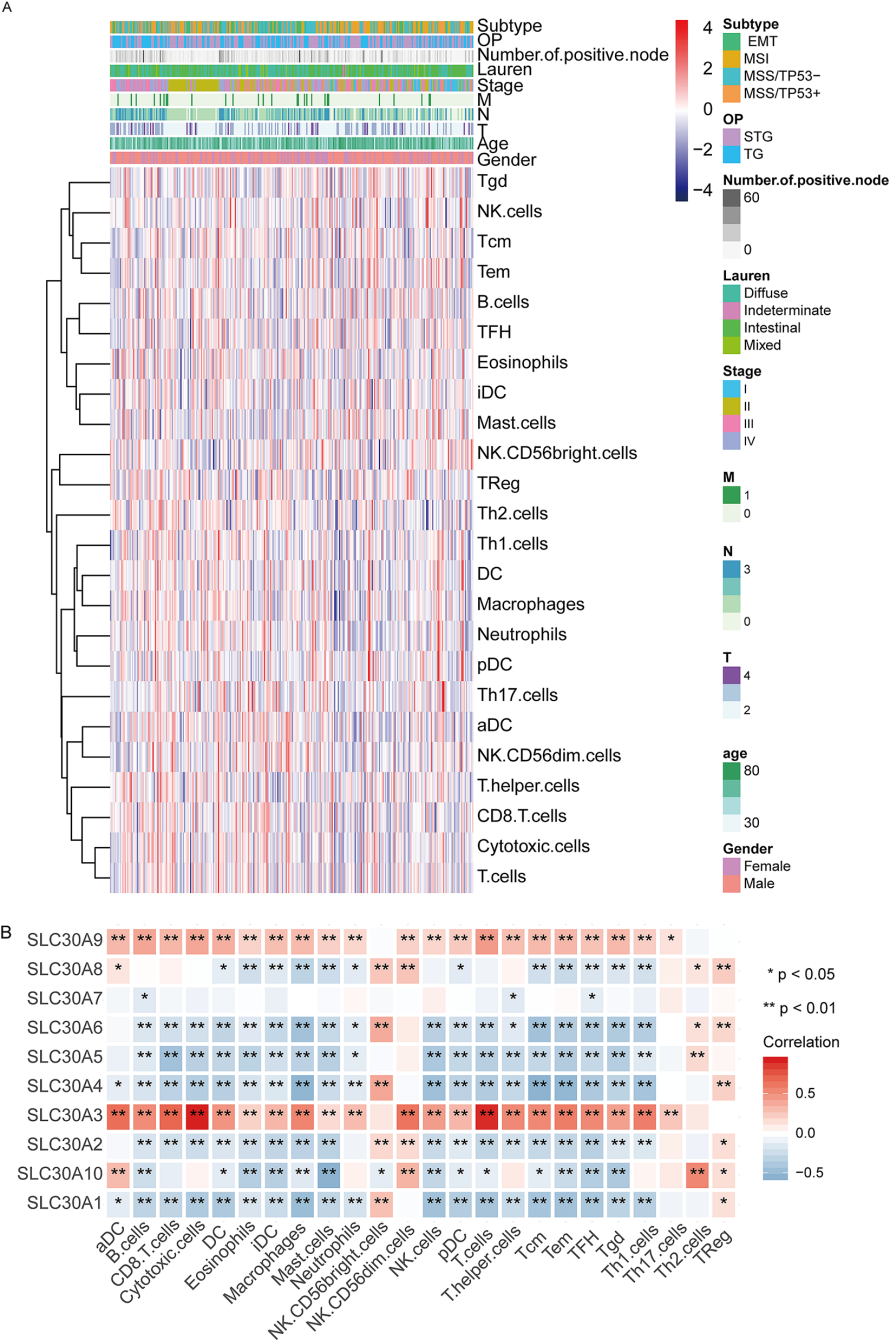
Immune landscape of gastric cancer. (**A**) Unsupervised clustering of 300 patients from the GSE62254 cohort using single-sample gene set enrichment analysis scores from 24 immune cell types. Molecular subtype, post operation type, number of positive nodes, Lauren classification, stage, T, N, M, age, as well as gender stage were annotated in the higher panel. Hierarchical clustering was performed with Euclidean distance and Ward linkage. (**B**) SLC30A family genes were associated with immune-cell subset. Red boxes indicate positive correlation and blue boxes indicate negative correlation. *, P < 0.05; **, P < 0.01.

The complex interactions between solid tumors and their microenvironment remain unclear, and previous studies had shown that immune infiltrates were significantly related to the progression and prognosis of GC^41–43^. We conducted the ssGSEA algorithm to deconvolve the relative abundance of each cell type based on expression profiling data obtained from GSE62254. The immune phenotype landscape as shown in Fig. 7A. We get further explored the coefficient of the association of SLC30A family genes in immune cell subsets (Fig. 7B). The results showed that SLC30A family genes were closely associated with the infiltration of immune cells, indicating that SLC30A family genes play an important role in GC partly because of immune infiltration.

## Discussion

In the present study, ROC analysis suggested that most SLC30A family genes had high diagnostic value for distinguishing GC patients from healthy individuals and could play an important role in GC diagnosis. Furthermore, univariate survival analysis showed that upregulated *SCL30A1*, *5–7*, and *9* expression was positively associated with favorable OS, FPS, and PPS. On the contrary, high expression of *SLC30A2-4*, *8*, and *10* were significantly correlated with poor OS, FPS, and PPS in GC patients. Moreover, all SLC30A family genes were strongly correlated with clinical characteristics. Taken together, all members of the SLC30A gene family could be utilized as promising prognostic biomarkers in GC patients.

Zinc is an indispensable trace element that is crucial for the proper function of various cellular proteins and essential for key physiological processes including nucleic acid metabolism, regulation of gene expression, cell division^44,45^. Furthermore, cancer cells may extract zinc from circulation to promote cancer growth^46,47^. In this study, to our best knowledge and for the first time, we used various large database, including TCGA, GEO, UALCAN, cBioPortal, STRING, and Kaplan–Meier Plotter, to systematically analyzed the expression level of SLC30A family genes, prognostic values, genetic alterations, and functional enrichment analysis in GC patients.

Aberrant zinc expression levels and regulation of SLC30A family genes have been reported in various kinds of cancer. *SLC30A1* is upregulated in bladder cancer and negatively targeted by miR-411 to inhibit the growth and metastasis of bladder cancer cells^48^. Upregulated *SLC30A1* expression of could lead to cytotoxic cell death in human ductal adenocarcinoma cell lines^49^. Meanwhile, SLC30A1 has high expression in ovarian cancer (OC) cell lines and tissues and a recovery experiment revealed that upregulated *SLC30A1* counteracts the effect of miR-8073 mimics on OC cell proliferation and apoptosis to affect the malignant progression of OC^50^. *SLC30A2* is dysregulated in breast cancer lines and *SLC30A2*-mediated Zn accumulation in mitochondria is associated with increased mitochondrial oxidation^51^. Meanwhile, *SLC30A2* over-expression leads to Zn vesicularization, shifts in cell cycle, enhanced apoptosis, and reduced proliferation and invasion in breast cancer^52^. *SLC30A2*-overexpression represses the cytotoxic effects of zinc hyper-accumulation in malignant metallothionein-null T47D breast tumor cells^53^. *SLC30A4* is significantly overexpressed in prostate cancer compared to normal tissues from other organs^22^. *SLC30A5-7* and *9* are significantly upregulated in colorectal cancer and *SLC30A9* is involved in the canonical Wnt pathway^24^. Overexpressed *SLC30A7* in esophageal squamous cell carcinoma could be a mechanism adapted by tumor cells to maintain the basal zinc requirement for carrying out vital functions during zinc deficiency^54^. *SLC30A7* is also significantly upregulated in hepatocellular carcinoma^55^. *SLC30A8* is aberrantly expressed in breast cancer and glioblastoma tumors, and decreased expression of SLC30A8 could contribute to the uncontrolled growth, proliferation, and tumor maintenance of glioblastoma multiforme cells^56,57^. SLC30A9 expression is significantly higher in hepatocellular carcinoma tissues than adjacent non-cancerous tissues, but is not correlated with survival in hepatocellular carcinoma patients^58^. *SLC30A10* is aberrantly expressed in colorectal cancer and is significantly related to the methylation epigenotype and molecular genesis of colorectal cancer^59,60^. In the present study, mRNA expression of *SLC30A1-3*, *SLC30A5-7*, and *9* was significantly upregulated in gastric cancer tissues compared to non-cancer tissues in GC patients, while *SLC30A4* was downregulated in cancer tissues.

To further clarify the genetic alteration and carcinogenic mechanism of SLC30A family genes, we found that the percentages of genetic alterations in SLC30A family genes for GC varied from 1.1 to 7% for individual genes. Furthermore, the results of Kaplan–Meier plotter and log-rank test showed no significantly statistical differences in OS and DFS in cases with and without SLC30A family gene alterations. Consistent with previous research, GO term analysis and KEGG pathway enrichment analysis showed that SLC30A family genes contributed to mineral transport related biological processes, including zinc transport, cellular zinc homeostasis, cellular transition metal ion homeostasis, and the mineral absorption pathway and our results showed that SLC30A family genes were closely associated with the infiltration of immune cells,. Therefore, we hypothesized that the action mechanism of SLC30A family genes induced tumorigenesis and progression by regulating zinc homeostasis in tumor cells and partly because of immune infiltration. This may provide a new insight in diagnosis and treatment of GC patients, especially in areas with zinc deficiency such as Cixian and Linxian.

## Conclusions

In conclusion, SLC30A family genes were aberrantly expressed in GC tissues. High expression of *SLC30A1*, *5–7*, and *9* as well as low expression of *SLC30A2-4*, *8*, and *10* were significantly associated with favorable prognosis in GC patients. High *SLC30A2* expression was significantly correlated with poor OS, FPS, and PPS in in all of GC patients indicating that these genes play an oncogenic role in GC and are markers for improved GC survival and prognostic accuracy.

## Supplementary information

Supplementary Information.

## Data Availability

Publicly available datasets were analyzed in this study. These data can be found here: TCGA, UALCAN, cBioPortal, and Kaplan–Meier Plotter.

## Abbreviations

GC: Gastric cancer
OS: Overall survival
FPS: First progression survival
PPS: Post progression survival
SLC30A: The solute carrier 30
TCGA: The Cancer Genome Atlas
FU: Fluorouracil
HER2: Human epidermal growth factor receptor 2
ssGSEA: Single-sample gene set enrichment analysis
