# Supplementary material for: Comprehensive analysis of the expression of SLC30A family genes and prognosis in human gastric cancer
Source: Sci Rep. 2020 Oct 27;10:18352. doi: 10.1038/s41598-020-75012-w (PMC7591519; doi:10.1038/s41598-020-75012-w)
Supplement: Supplementary file 1 — Supplementary Information. [file 41598_2020_75012_MOESM1_ESM.docx]

**Comprehensive analysis of the expression of SLC30A family genes and prognosis in human gastric cancer**

**Yongdong Guo^1^, Yutong He^1^**

**1 Cancer Institute, Fourth Hospital of Hebei Medical University, Shijiazhuang, 050011, China**

**Correspondence should be addressed to: Yutong He, (E-mail: 15733291685@163.com)**

**Short Title: SLC30A genes as predictive gastric cancer markers**

**Supplementary materials:**

**
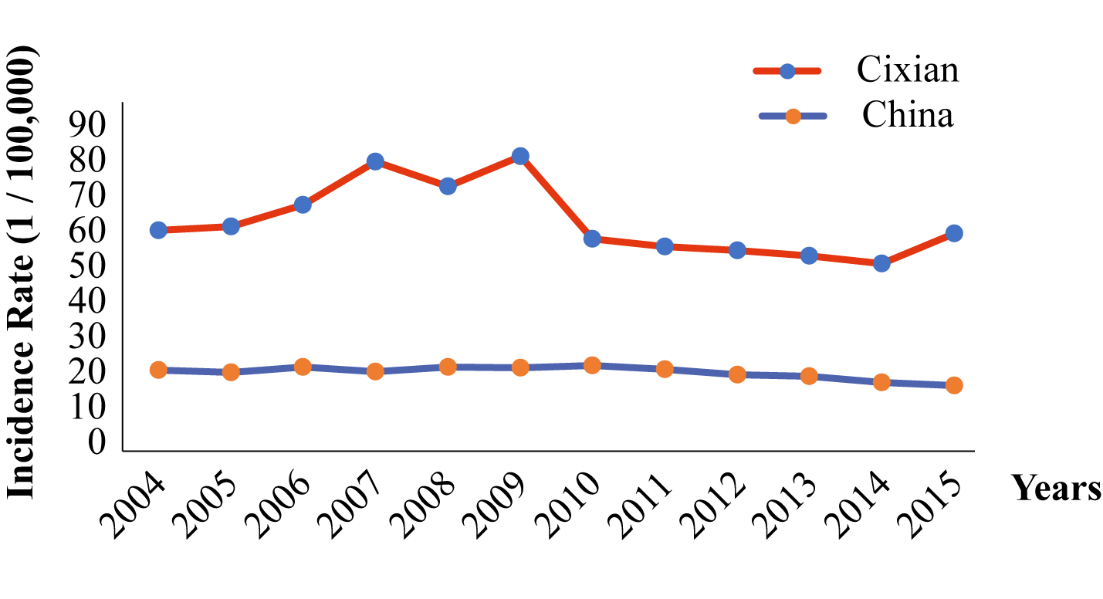
**

**Figure S1.** Incidence rate of gastric cancer in Cixian and China from 2004 to 2015 (World Standard Rate). Incidence rate data of gastric cancer in Cixian and China obtained from Hebei Cancer Registry Annual Report and Chinese Cancer Registry Annual Report from 2004 to 2015, respectively.

**
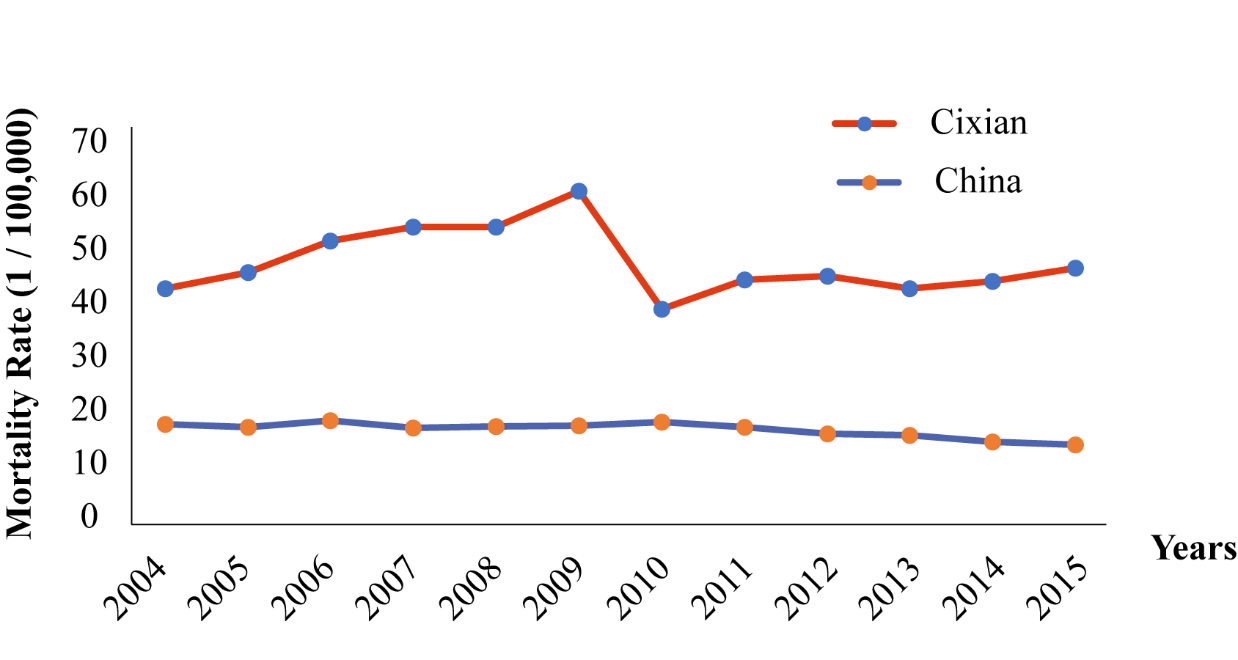
**

**Figure S2.** Mortality rate of gastric cancer in Cixian and China from 2004 to 2015 (World Standard Rate). Mortality rate data of gastric cancer in Cixian and China obtained from Hebei Cancer Registry Annual Report and Chinese Cancer Registry Annual Report from 2004 to 2015, respectively.

**
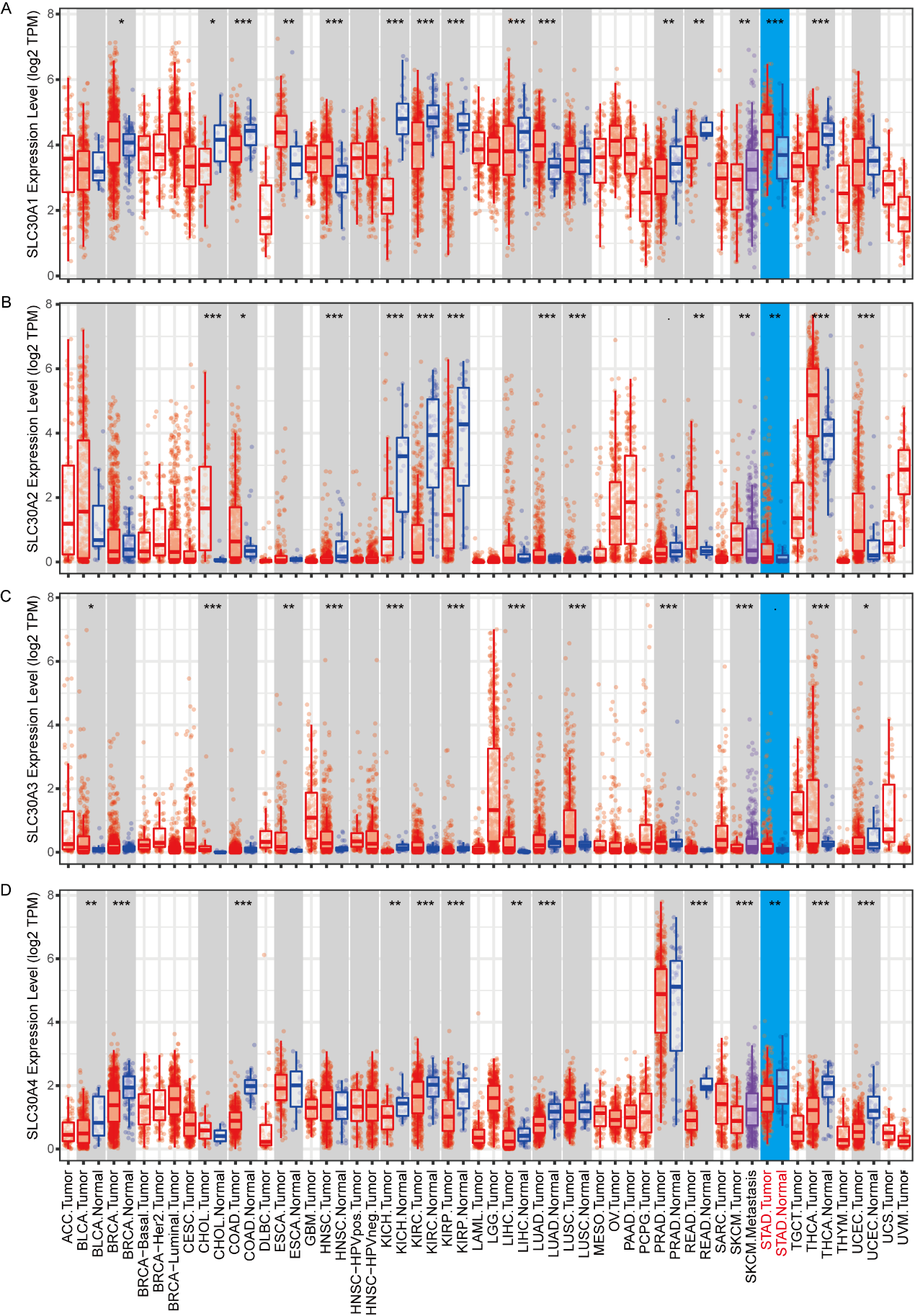
**

**
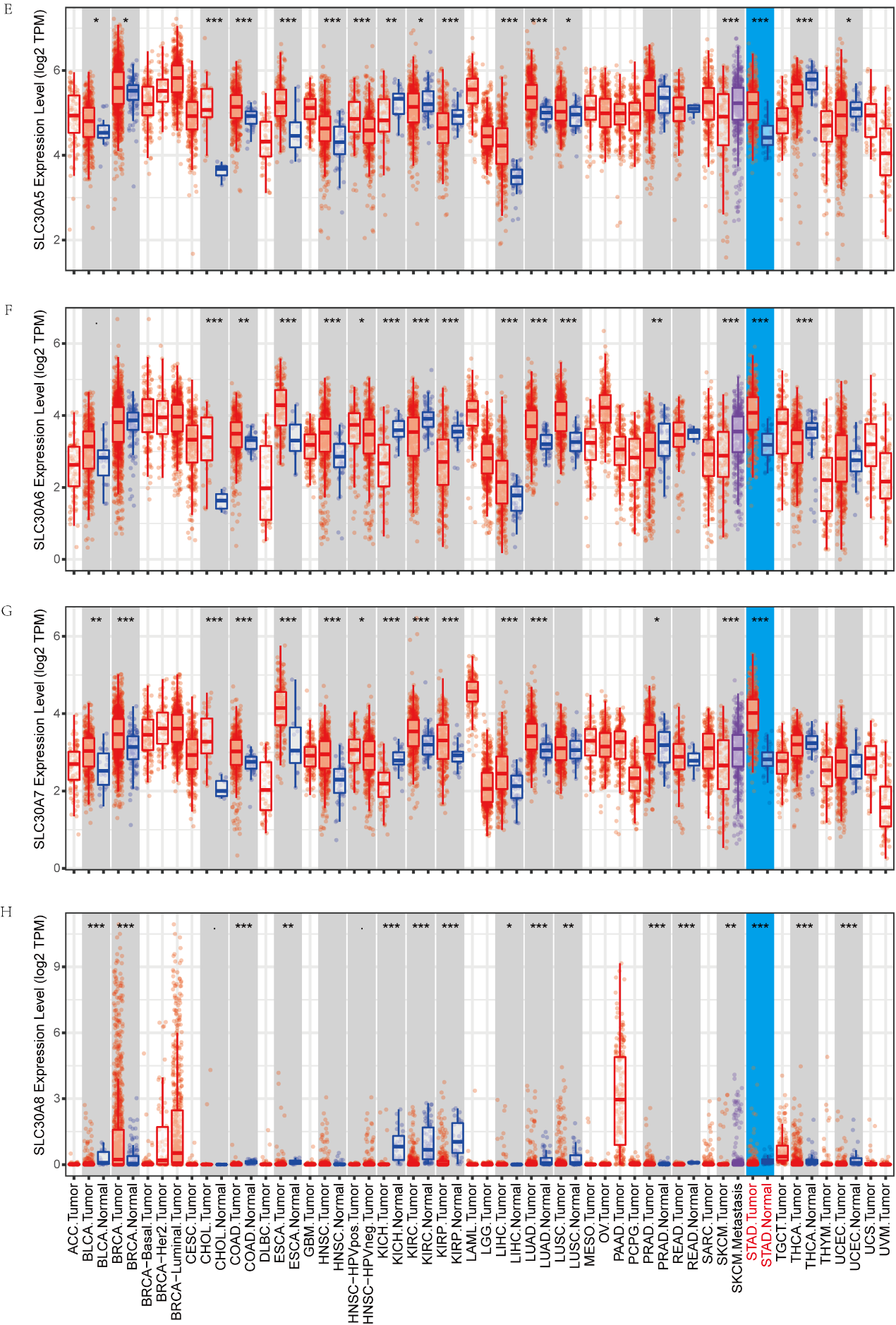
**

**
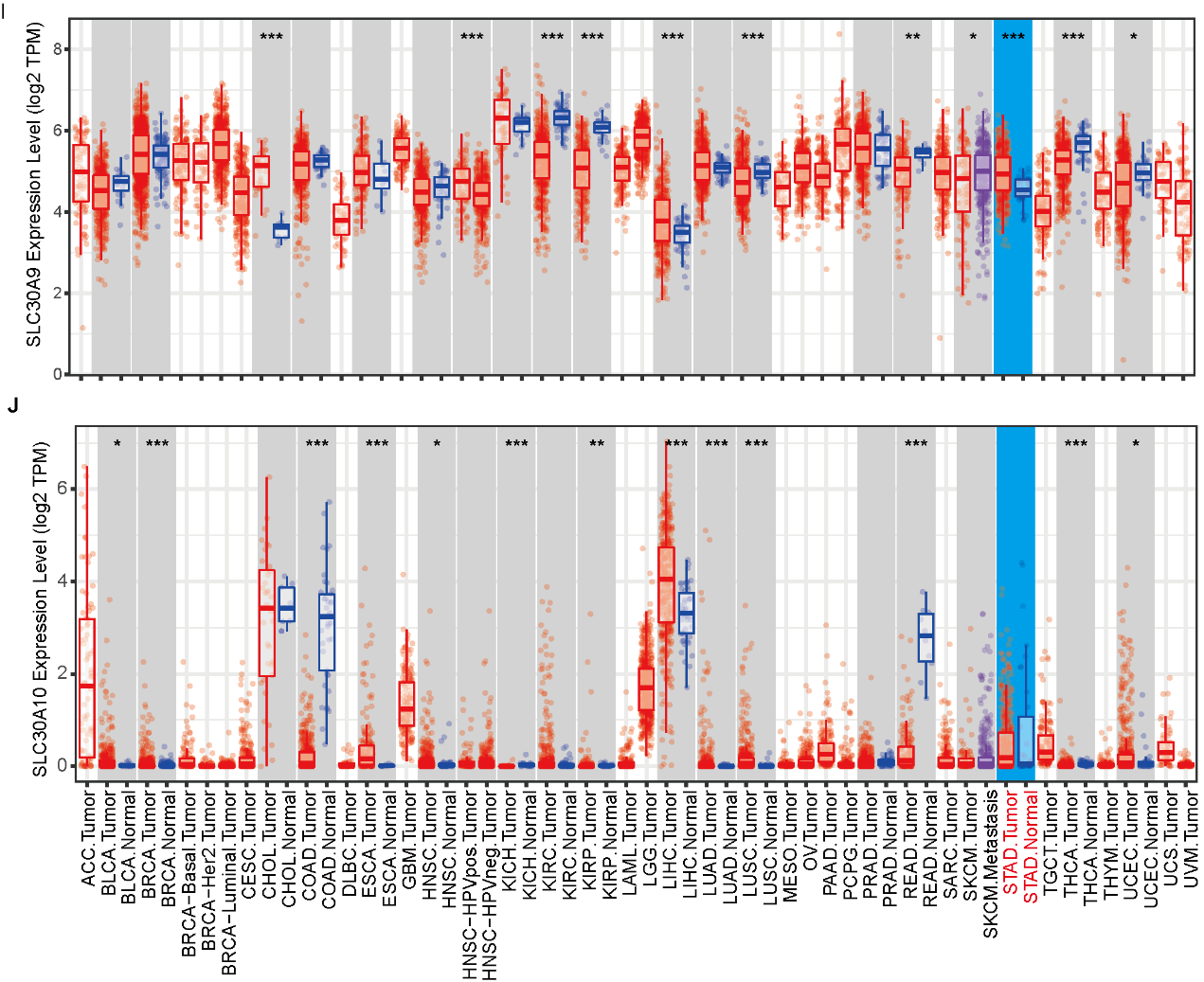
**

**Figure S3.** SLC30A family genes expression profile across all tumor samples and normal tissues analyzed by TIMER database. *: *P*<0.05, **: *P*<0.01, ***: *P*<0.001, NS: *“”* >0.05.

**Table S1**. Clinical features of patients with GC (n = 40).

| **Clinicopathological parameters** | **Group** | **Number of cases (%)** |
| --- | --- | --- |
| **Sex** | Male | 29 (72.5%) |
|  | Female | 11 (27.5%) |
| **Age (years)** | ≥65 | 18 (45.0%) |
|  | <65 | 22 (55.0%) |
| **Tumor size (cm)** | <5 | 24 (60.0%) |
|  | ≥5 | 16 (40.0%) |
| **Histologic differentiation** | Well or moderate | 12 (30.0%) |
|  | Poor | 28 (70.0%) |
| **TNM stage** | Ⅰ+Ⅱ | 19 (47.5%) |
|  | Ⅲ‐Ⅳ | 21 (52.5%) |
| **Distant metastasis** | Absent | 17 (42.5%) |
|  | Present | 23 (57.5%) |

**Table S2.** The primers of SLC30A family genes and GAPDH

| **Gene** |  | **Sequence** |
| --- | --- | --- |
| **GAPDH** | **F** | 5′-CCACCCATGGCAAATTCC-3′ |
|  | **R** | 5′-GATGGGATTTCCATTGATGACA-3′ |
| **SLC30A1** | **F** | 5‘- GCATCAGTTTATGAGGCTGGTCCT -3’ |
|  | **R** | 5’ CAGGCTGAATGGTAGTAGCGTGAA -3’ |
| **SLC30A2** | **F** | 5’-TTCTGTCTGTCCACATCGCC-3’ |
|  | **R** | 5’-AGAAAGTTGGGCAGTCTGAGG-3’ |
| **SLC30A3** | **F** | 5‘-ACCATGTTGCCTCTGCACAC-3’ |
|  | **R** | 5’-CATCTCCGGCTGATACTGCTC-3’ |
| **SLC30A4** | **F** | 5’-CCTAATAGGTGTGCCAAGCCA-3’ |
|  | **R** | 5’- CAACCCCAGGCCCACATTTA-3’ |
| **SLC30A5** | **F** | 5'-TCAAAAGCCATTTTCTTCTGGG-3' |
|  | **R** | 5'-ACAAGAAAGTTGGGCAGTTTCC-3' |
| **SLC30A6** | **F** | 5'-GGAGTTCCAGGAATTGGAGCA-3' |
|  | **R** | 5'-AAGAAAGTTGGGCATGGTCTTG-3' |
| **SLC30A7** | **F** | 5'-GAGCAGCCTACGGTTCCTCAA-3' |
|  | **R** | 5'-TGCAGAGTGCCAACCTCCTCT-3' |
| **SLC30A8** | **F** | 5'-GCCAAGTGGTTCGGAGAGAA-3' |
|  | **R** | 5'-GAAAGTTGGCAAGTCACAGGG-3' |
| **SLC30A9** | **F** | 5’-GCTCCACTTA AGCAAGAACCT-3’ |
|  | **R** | 5’-TCGCTCTGACTC CAGTGATGAA-3’ |
| **SLC30A10** | **F** | 5’-AGCCTGACTGTCCTCATGGT-3’ |
|  | **R** | 5’-TAATGCTTGTCCTTTGGCCTG-3’ |

**Table S3.** Clinicopathological characteristics of valid patients with gastric cancer in Kaplan-Meier plotter database.

| **Characteristics** | **Group** | **Number (%)** |
| --- | --- | --- |
| **Gender** | Male | 349 (65.1%) |
|  | Female | 187 (34.9%) |
| **HER2** | Positive | 202 (32.0%) |
|  | Negative | 429 (68.0%) |
| **Stage** | Ⅰ | 62 (11.5%) |
|  | Ⅱ | 140 (26.0%) |
|  | Ⅲ | 197 (36.5%) |
|  | Ⅳ | 140 (26.0%) |
| **Lauren classification** | Intestinal | 269 (50.0%) |
|  | Diffuse | 240 (44.6%) |
|  | Mixed | 29 (5.4%) |
| **Differentiation** | Poorly | 121 (62.7%) |
|  | Moderately | 67 (34.7%) |
|  | Well | 5 (2.6%) |
| **Treatment** | Surgery alone | 380 (77.5%) |
|  | 5 FU based adjuvant | 34 (7.0%) |
|  | Other adjuvant | 76 (15.5%) |
| **Perforation** | No | 169 (100%) |
